# Uranium Repartitioning during Microbial Driven Reductive Transformation of U(VI)-Sorbed Schwertmannite and Jarosite

**DOI:** 10.1021/acs.est.4c03645

**Published:** 2024-10-03

**Authors:** Changxun Yu, Anders Johnson, Andreas Karlsson, Roman Chernikov, Viktor Sjöberg, Zhaoliang Song, Mark Dopson, Mats E. Åström

**Affiliations:** † Department of Biology and Environmental Science, Linnaeus University, 39231 Kalmar, Sweden; ‡ Centre for Ecology and Evolution in Microbial Model Systems (EEMiS), Linnaeus University, 39231 Kalmar, Sweden; § Department of Geosciences, Swedish Museum of Natural History, 10405 Stockholm, Sweden; ∥ Canadian Light Source, 44 Innovation Boulevard, Saskatoon, SK S7N 2 V3, Canada; ⊥ Man-Technology-Environment Research Centre (MTM), Örebro University, 70182 Örebro, Sweden; # Institute of Surface-Earth System Science, School of Earth System Science, Tianjin University, 300072 Tianjin, China

**Keywords:** Uranium, acidic drainage, schwertmannite, jarosite, microbial communities, X-ray absorption spectroscopy

## Abstract

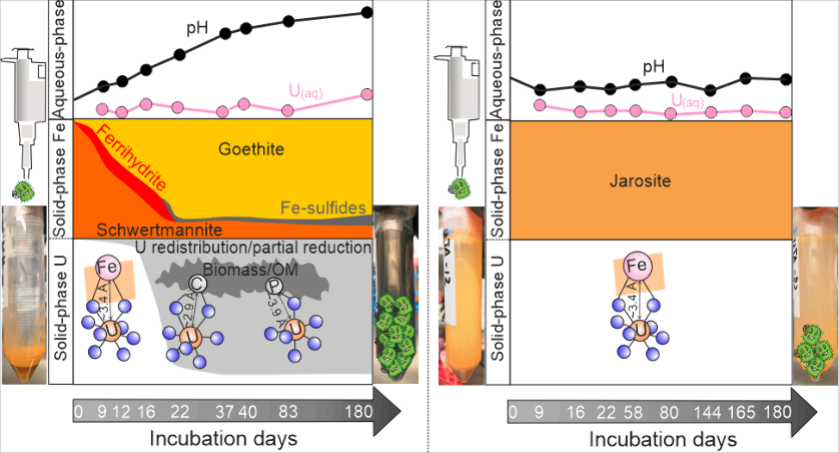

This study exposes U(VI)-sorbed schwertmannite and jarosite to biotic reductive incubations under field-relevant conditions and examines the changes in aqueous and solid-phase speciation of U, Fe, and S as well as associated microbial communities over 180 days. The chemical, X-ray absorption spectroscopy, X-ray diffraction, and microscopic data demonstrated that the U(VI)-sorbed schwertmannite underwent a rapid reductive dissolution and solid-phase transformation to goethite, during which the surface-sorbed U(VI) was partly reduced and mostly repartitioned to monomeric U(VI)/U(IV) complexes by carboxyl and phosphoryl ligands on biomass or organic substances. Furthermore, the microbial data suggest that these processes were likely driven by the consecutive developments of fermentative and sulfate- and iron- reducing microbial communities. In contrast, the U(VI)-sorbed jarosite only stimulated the growth of some fermentative communities and underwent very limited reductive dissolution and thus, remaining in its initial state with no detectable mineralogical transformation and solid-phase U reduction/repartitioning. Accordingly, these two biotic incubations did not induce increased risk of U reliberation to the aqueous phase. These findings have important implications for understanding the interactions of schwertmannite/jarosite with microbial communities and colinked behavior and fate of U following the establishment of reducing conditions in various acidic and U-rich settings.

## Abstract 2

Our data reveal a divergent repartitioning of U during microbial driven reductive transformation of U(VI)-sorbed schwertmannite and jarosite

## 1 Introduction

Weathering of sulfide-containing soils, bedrock, and mining wastes at numerous sites worldwide releases large amounts of acidic sulfate-rich drainages with elevated levels of the highly carcinogenic and toxic radionuclide uranium (U) into the surrounding ecosystems.^1−8^ For instance, shallow groundwater in areas with natural and mined black shale (a common sulfidic rock containing up to several hundred mg/kg U^9^) in Sweden and Germany is frequently loaded with up to ∼400 and ∼3400 μg/L U, respectively.^2,5^ Similarly, acidic drainage discharged from waste rock piles of an abandoned U mine in South China contains ∼1400 μg/L U.^1^ Besides these sulfidic U-rich materials, the weathering of acid sulfate soils developing on sulfidic sediments result in discharge of considerable amounts of U into recipient watercourses.^2,4^

The release of acidic sulfate-rich drainage often leads to the formation of massive schwertmannite (Fe_8_O_8_(OH)_8–2*x*_(SO_4_)_*x*_·nH_2_O, where 1 < *x* < 1.75) and jarosite (MFe_3_(SO_4_)_2_(OH)_6_, where M is K^+^, Na^+^, NH_4_^+^, or H_3_O^+^). These Fe oxyhydroxysulfates act as key players in the cycling of Fe, S, carbon, contaminants, and nutrients in various acidic settings.^10−16^ Compared to schwertmannite, jarosite typically forms at lower pH (e.g., pH < 3)^10,15^ and has a higher crystallinity but lower surface area,^15,17^ making it more stable under natural conditions.^18^ Given the relatively large reactive surfaces of these two Fe oxyhydroxysulfates (1.5–7.5 m^2^/g),^15,17^ they can act as crucial scavengers for U in acidic drainages. Indeed, previous batch experiments showed that 200 mg of schwertmannite can adsorb nearly all the dissolved U(VI) species in 1 L acidic solutions (pH = 4.2) containing 40/45 μM U(VI) under a N_2_ atmosphere within 24 h.^19^ However, these Fe oxyhydroxysulfates are unstable and can undergo reductive dissolution/transformation when they are buried in benthic sediments, or subjected to prolonged waterlogged conditions due to changes in the hydrological regime as a result of future climate change, land-use change or remediation activities.^12,20,21^ Therefore, these Fe oxyhydroxysulfates and linked reductive transformation processes may act as key controls on the migration and environmental fate of U in acidic settings. Yet, there is no research examining how the reductive transformation of Fe oxyhydroxysulfates influences the mobility, speciation, and repartitioning of coassociated U.

Microorganisms are key biological factors driving the reductive dissolution/transformation of schwertmannite and jarosite. Incubation experiments show that natural consortia of anaerobic microorganisms trigger and sustain a rapid and efficient reductive transformation of schwertmannite under acidic conditions.^12,22−24^ However, these studies did not characterize the evolution of microbial community composition, impeding the ability to fully understand the key biogeochemical processes/pathways underlying the reductive transformation of schwertmannite. Although the reductive dissolution and solid-phase transformation of jarosite have been well-documented experimentally, these experiments were conducted either (i) in the presence of Fe- or sulfate-reducing enrichment cultures under (near-)neutral pH conditions (pH = 6.5–7.4),^25−33^ (ii) in natural acidic sediments/soils amended with synthetic jarosite,^24,34,35^ or (iii) abiotically under relatively high Fe(II)_(aq)_ and pH conditions (e.g., pH > 5.5).^36,37^ Thus, it remains unknown if and how natural microbial communities themselves trigger the reductive transformation of jarosite under low-pH conditions.

Here, incubation experiments were conducted with U(VI)-sorbed schwertmannite and U(VI)-sorbed jarosite that were mixed with artificial acidic groundwater and subsequently exposed to a natural consortium of anaerobic microorganisms. The aims were to explore (i) whether and how natural microbial communities evolve and drive the reductive transformation of these two oxyhydroxysulfates and (ii) how these processes impact the mobility, redox status, and repartitioning of coassociated U.

## 2 Materials and Methods

### 2.1 Mineral Synthesis and Characterizations

U(VI)-sorbed schwertmannite and jarosite were aseptically prepared by reacting freshly synthesized jarosite and schwertmannite with 2 L of 0.21 mM U(VI) at pH 4.5 (for details, see Text S1, Supporting Information). The resulting precipitates were analyzed in triplicate for the total concentrations of K, Fe, and U by inductively coupled plasma mass spectrometry (ICP-MS) and for Brunauer–Emmett–Teller (BET) surface areas using a Micromeritics ASAP 2020 surface area analyzer.

### 2.2 Incubation Experiments and Chemical Analyses

Incubation experiments were performed with the U(VI)-sorbed schwertmannite and U(VI)-sorbed jarosite, following the same procedures under aseptic conditions. Briefly, 0.8 g of U(VI)-sorbed mineral (jarosite or schwertmannite) were mixed with 40 mL of artificial groundwater in 50 mL Falcon tubes. The groundwater contained 5 mM CaSO_4_, 1 mM KCl, 5 mM MgSO_4_, 1 mM Na_2_SO_4_, 0.1 mM KH_2_PO_4_, 1 mM Na_4_SiO_4_, 1 mL/L Wolfe’s mineral solution,^38^ 1 g/L yeast extract, 0.5 g/L Aldrich humic acid, and 2 g/L glucose and then adjusted to pH 3.5 with diluted H_2_SO_4_. The groundwater mimicked the composition of natural groundwater in typical black shale and acid sulfate soil settings.^39^ Each of the mineral-groundwater suspensions (except the abiotic controls in the middle and at the end of each experiment) were inoculated with a natural consortium of anaerobic microorganisms by adding 500 μL of 1:10 sediment:water suspension prepared from fresh anoxic sediment from an acidic ditch draining a hemiboreal acid sulfate soil.^4^ The tubes with mineral-groundwater suspensions were transferred to an anaerobic chamber (O_2_ < 0.5 ppm) and allowed to equilibrate with the O_2_-free atmosphere for 6 h, after which they were tightly capped and sealed. The suspensions were incubated at room temperature for 9 to 180 days with regular gentle shaking inside the anaerobic chamber. At each sampling time, triplicate suspensions were withdrawn and centrifugated at 12000 g for 15 min to separate solid and aqueous phases. The obtained solutions were filtered through 0.45 μm syringe filters and immediately analyzed for pH, as well as the concentrations of Fe(II) and total Fe using the 1,10 phenanthroline method.^40^ The remaining solutions were analyzed for total concentrations of U, S, U, P, Ca, K, Mg, Na, and Si by ICP-MS or inductively coupled plasma optical emission spectroscopy. Portions of the solid phases were immediately frozen and stored at −22 °C for microbial community analyses, while the remaining materials were dried and stored under O_2_-free conditions.

### 2.3 SEM-EDS and XRD

The morphology and chemical compositions of the solid phases at the beginning and the end of the incubation experiments were examined on an FEI (now Thermo-Scientific) QUANTA FEG 650 Scanning Electron Microscope (SEM) fitted with an 80 mm^2^ Oxford X-Max Energy Dispersive Spectrometry (EDS). The samples were dried on thin glasses in an anaerobic chamber. The analyses were performed in a low-vacuum (1 mbar) environmental mode, which allows the use of uncoated samples and thus, EDS analyses of carbon-bearing phases.

Mineralogical changes during the two incubation experiments were determined by X-ray diffraction (XRD) using a PANalytical X’Pert3 Powder diffractometer system (Cu Kα1-radiation) fitted with an X’Celerator strip detector. The initial schwertmannite (USCH) and jarosite (UJA) as well as solid-phase samples withdrawn at selected times (referred to as mineral acronym + day of withdrawal) were scanned from 5° to 70° 2θ for 20 min. The XRD patterns were evaluated using the PANalytical High-Score Plus software (V.4.7).

### 2.4 Fe and U X-ray Absorption Spectroscopy

The speciation of Fe and U in the USCH and UJA as well as selected solid-phase samples were examined via X-ray absorption spectroscopy (XAS) on the BioXAS-Main beamline at the Canadian Light Source. The XAS data at the Fe K-edge were collected in transmission mode, while those at the U L_3_-edge in fluorescence mode using a 32-element Ge solid-state detector. Energy calibration was performed by simultaneously recording the XAS spectrum of a Fe and Y foil, respectively. The scan sizes for the pre-edge, XANES, and EXAFS regions were 10 eV/step, 0.5 eV/step, and 0.05 Å^–1^/step, respectively. The reduction of XAS data was performed using the Athena program,^41^ following the standard procedures (e.g., energy calibration, background removal, normalization). To quantify the solid-phase Fe speciation and U oxidation state in the samples, linear combination fitting (LCF) was performed on the Fe EXAFS spectra and U XANES spectra, respectively (details are given in Text S2). The U EXAFS spectra were further converted to R-space via Fourier transforms (FT) over the *k* range of 3.3 to 11.5 or 12 Å^–1^ using a Hanning window. The resulting spectra for each sample (with *k*-weighting of 1, 2, 3) were fitted simultaneously in R-space (R = 1–3.35 to 3.77 Å) using the Artemis program^41^ (for details, see Text S2), to obtain quantitative structural information about the local environment around the U atoms in the samples.

### 2.5 Microbial Analyses

Details of the DNA extraction, 16S rRNA gene PCR amplification, amplicon sequencing, and bioinformatics are provided in Text S3. Briefly, DNA extractions were performed using the Qiagen DNeasy Powersoil followed by PCR amplification to generate 2 × 300 bp pair-end reads^42^ for sequencing on the Illumina MiSeq platform.^43^ Sequencing reads were processed using the Ampliseq pipeline,^44^ which incorporates QIIME^45^ and DADA2^46^ annotated against the SILVA database v138.1.^47^ Downstream processing was performed in R and RStudio,^48^ using the packages vegan^49^ and tidyverse.^50^

## 3 Results

### 3.1 Characteristics of the Synthetic U(VI)-Sorbed Schwertmannite and Jarosite

The mineralogy of the USCH and UJA was confirmed by XRD (Figure S1a, b). The USCH had a BET surface area of 4.1 m^2^/g and contained 39.8 ± 0.7% Fe and 2689.3 ± 97.2 mg/kg U, while the UJA had a BET surface area of 1.9 m^2^/g and contained 30.0 ± 1.3% Fe, 5.3 ± 0.3% K, and 290.1 ± 10.1 mg/kg U. These results agreed with previous reports for the jarosite and schwertmannite group minerals.^17,26,51^

**Figure 1:**
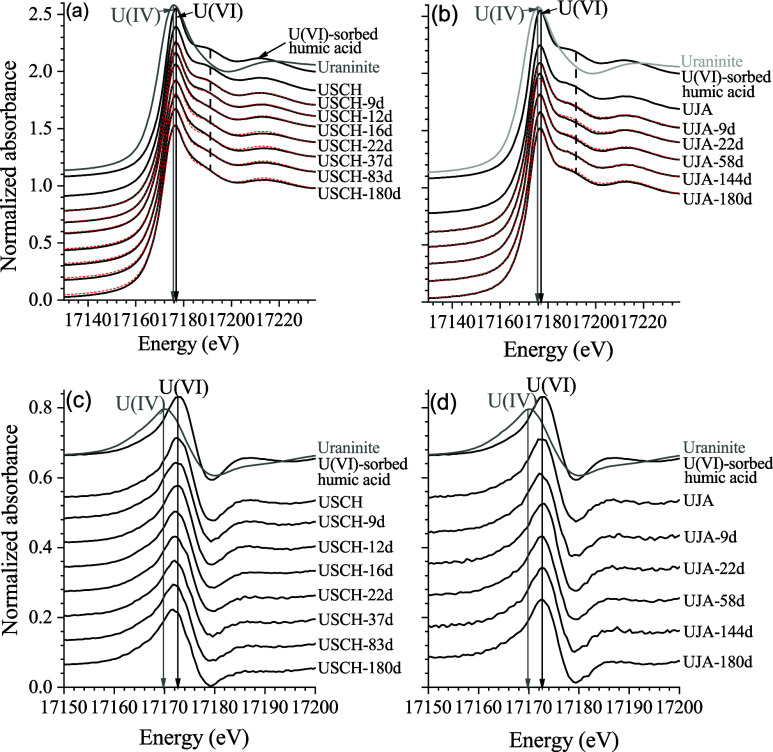
Uranium L3 edge XANES spectra (a, b) and their first derivatives (c, d) for the initial U(VI)-sorbed schwertmannite (USCH) and jarosite (UJA) plus selected solid-phase samples for the two incubation experiments. The spectra of uraninite and U(VI)-sorbed humic acid are shown for comparison. The gray or black solid lines are experimental spectra, while red dashed lines represent the LCF-XANES fits. The dashed vertical black lines in (a) and (b) mark the central position for the resonance feature arising from the tightly bound axial oxygen atoms of uranyl. The vertical gray and black arrows mark the position of U(IV) and U(VI), respectively.

**Figure 2:**
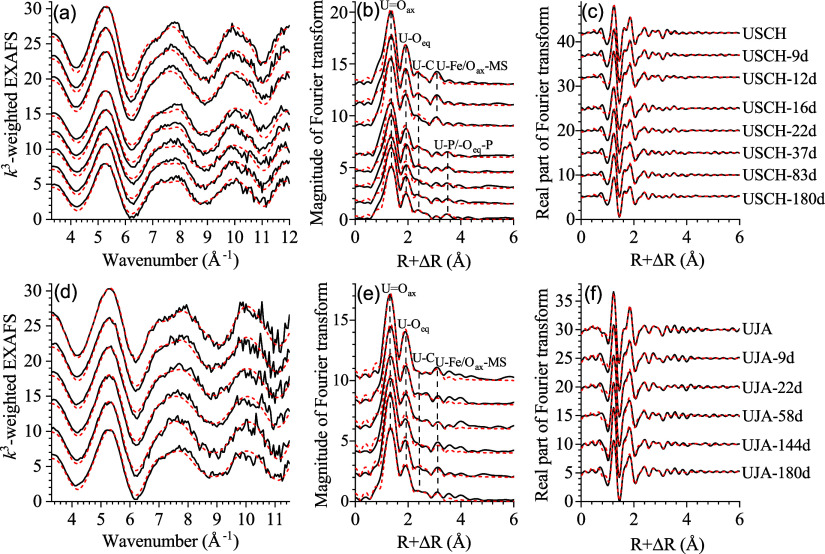
U L3-edge EXAFS spectra as well as their Fourier-transform magnitudes and real parts for solid-phase samples obtained from the incubation experiments with U(VI)-sorbed schwertmannite (a–c) and U(VI)-sorbed jarosite (d–f). Experimental data are shown as solid black lines, while the fits as dashed red lines. The structural parameters of the fits are given in Table S1. USCH and UJA are the initial U(VI)-sorbed schwertmannite and U(VI)-sorbed jarosite, respectively, used for the two incubation experiments. Vertical dashed lines in (b) and (e) mark the contributions from different scattering paths.

The U L_3_-edge XANES spectra for USCH and UJA displayed characteristic features for U(VI), including a shift of the absorption edge/peak toward higher energies relative to the uraninite standard (+IV) and the appearance of a pronounced resonance feature (∼17191 eV) arising from the axial O atoms of uranyl (Figure 1). Also, the U L_3_-edge EXAFS spectra were similar for the two minerals (Figure 2), pointing to a similar initial local binding environment of the adsorbed U(VI). In particular, the oscillation peaks at the *k* = ∼6.5–8.5 Å^–1^ were split, indicating the presence of uranyl carbonate species.^52,53^ In accordance with these results, the EXAFS spectra and corresponding major FT peaks were well-reproduced by a structural model (Figure 2) where U(VI) binds to the Fe sites on the schwertmannite and jarosite surfaces. The U–C and U–Fe distances (Table S1) were typical for ternary ≡FeO_surface_-U(VI)-carbonate/bicarbonate complexes where U(VI) is bound to Fe and C through two equatorial O atoms, respectively, in a bidentate fashion.^52−56^

### 3.2 Aqueous-Phase Chemistry

**Figure 3:**
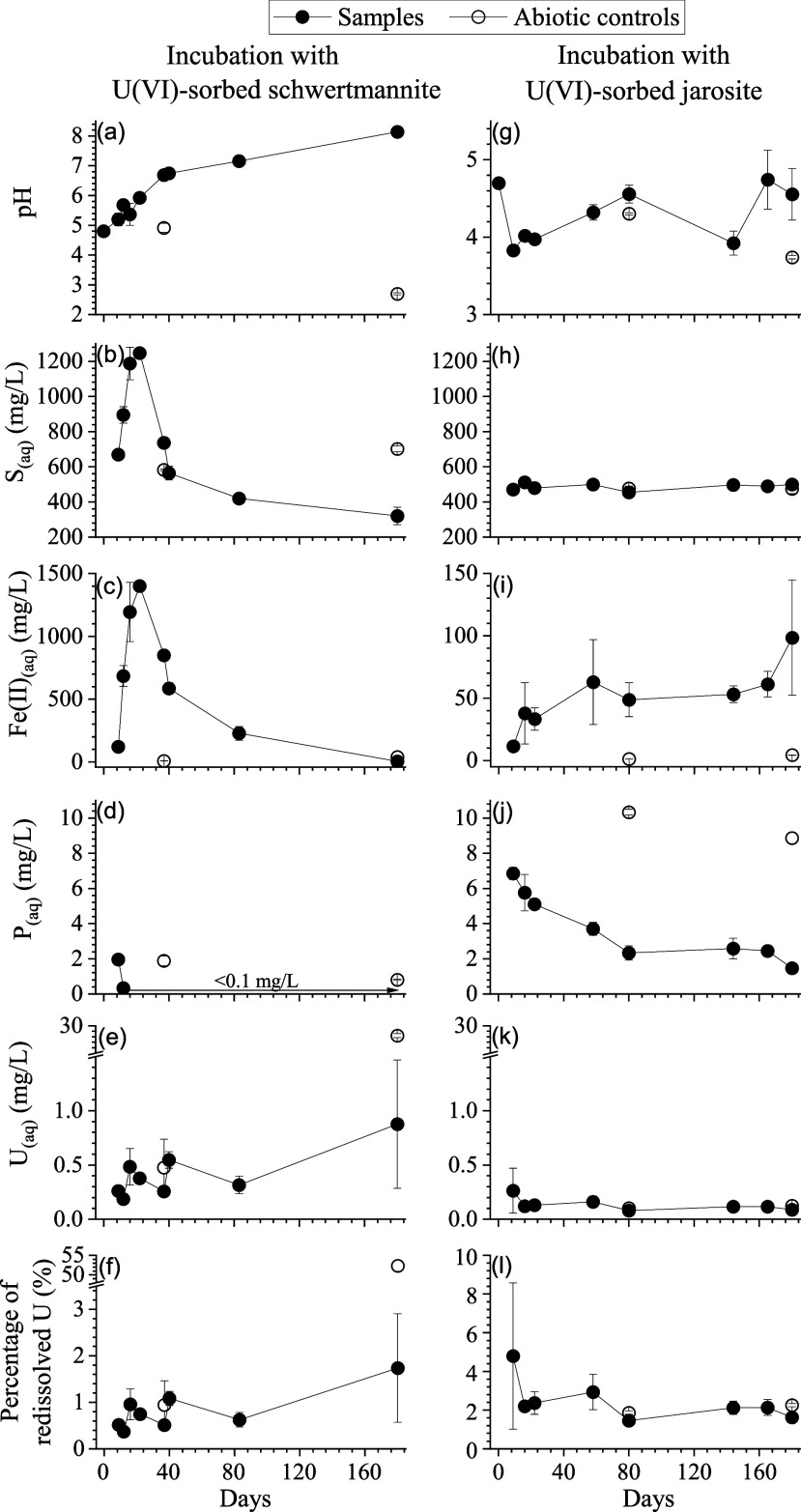
Temporal evolution of pH, concentrations of selected aqueous-phase components, and percentages of redissolved U for the incubation experiments with U(VI)-sorbed schwertmannite and U(VI)-sorbed jarosite over 183 days. Data points are the averages ± standard deviation of triplicate samples.

During the USCH incubation, the pH of the aqueous phases steadily increased from 4.8 to 8.1 ± 0.2, in sharp contrast to the abiotic controls for which the pH decreased to 2.7 ± 0.1 (Figure 3a, Table S2). The concentrations of both Fe(II)_(aq)_ and S_(aq)_ were characterized by a strong increase over the first 22 days, followed by a steep decrease between 22 and 44 days after which the concentrations continued to decline but at lower rates toward the end of the experiment (Figure 3b, c, Table S2). The concomitant decrease in Fe(II)_(aq)_ and S_(aq)_ after day 22 was accompanied by the appearance and buildup of black precipitates on the tube walls and the surface of the solids, which were not observed in the abiotic controls. The concentrations of P_(aq)_ decreased rapidly to ∼0.33 ± 0.11 mg/L by day 12 and then remained at <0.10 mg/L thereafter (Figure 3d, Table S2). The concentrations of U_(aq)_ were overall low but increased slightly from ∼0.3 ± 0.0 to 0.9 ± 0.6 mg/L during the experiment, accounting for only 1.7 ± 1.1% of the initial U load in the solids (Figure 3e, f and Table S2). In contrast, 52.3 ± 1.4% of the U load in the abiotic control solids was released to the aqueous phase by the end of the experiment.

The aqueous-phase properties of the UJA incubation experiment displayed much less change over the 180-day incubation (Figure 3g-l and Table S3). The pH and the concentrations of S_(aq)_ and K_(aq)_ fluctuated from 3.8 ± 0.0–4.7 ± 0.4, 454.3 ± 23.0–511.3 ± 17.8 mg/L, and 130.0 ± 2.2–163.7 ± 8.7 mg/L, respectively, generally falling within the ranges of the abiotic controls. Over time, the concentrations of Fe(II)_(aq)_ overall increased from 11.4 ± 1.4 to 98.5 ± 4.6 mg/L, whereas those of P_(aq)_ decreased from 6.9 ± 0.3 to 1.5 ± 0.1 mg/L. This was in sharp contrast to the controls, which exhibited consistently low Fe(II)_(aq)_ concentrations and high P_(aq)_ concentrations. The concentrations of U_(aq)_ remained very low throughout the incubation experiment (<0.3 ± 0.2 mg/L) and were similar as in the abiotic controls, accounting for less than 4.8 ± 3.8% of the initial solid-phase U load.

### 3.3 Mineralogical Transformations of Fe

The XRD analysis combined with the LCF-EXAFS results revealed a relatively rapid partial transformation of the USCH to ferrihydrite by day 9 and further to goethite by day 12 (Table 1; Figure S1a). The partial transformation of USCH was further confirmed by the SEM-EDS observations, showing that the solid phases on these 2 days were composed of subrounded and nanosized particles with limited compositional variations (Figures S6–S7). From day 16 onward, the weak and broad XRD peaks arising from schwertmannite were not invisible and replaced by intense diffraction peaks whose positions and relative intensities matched with those of goethite (Figure S1a). Similarly, the EXAFS spectra from day 16 onward displayed several notable changes that reflect the three characteristic FeO_6_ linkages in the goethite structure, including the presence of a small but distinctive peak at *k*=∼5.5 Å^–1^, a stronger peak between *k*=∼7.1–7.9 Å^–1^, and a splitting of the peak between 10.2 and 11.2 Å^–1^ (Figure S2). Accordingly, the LCF-EXAFS analysis predicted that the solid-phase Fe in these samples was dominated by goethite, along with small fractions of FeS and remaining schwertmannite (Table 1). At the end of the incubation, the solid phases also displayed a marked change in surface morphology and chemical compositions, consisting of large aggregates with strongly elevated molar Fe/S ratios, patched with S-rich nanoparticles and a few Fe–Ca carbonate crystals (Figure S8 and Table S4). In the abiotic control, in comparison, the SEM-EDS, XRD, and EXAFS data showed that schwertmannite largely persisted and only partially transformed to goethite at the end of the incubation (Table 1 and Figures S1, S9).

**Table 1:** Iron Speciation and U Oxidation State in Selected Solid-Phase Samples and Abiotic Controls (Marked with “C”) for the Two Incubation Experiments, Quantified by Linear Combination Fitting of k^3^-Weighted Fe EXAFS Spectra (*k* = 2-12 Å^–1^) and Normalized U XANES Spectra (17140–17240 eV), Respectively^a^

	Fe LCF-EXAFS				U LCF-XANES				
	Jarosite	Schwertmannite	2-line ferrihydrite	FeS	Goethite	R-factor^c^	U(VI)	U(IV)	R-factor^c^
USCH-9d		91	9			0.00172	97	3	0.00008
USCH-12d		71	13		16	0.00074	95	5	0.00011
USCH-16d		23	10	5	62	0.00306	84	16	0.00023
USCH-22d		23		5	72	0.00361	86	14	0.00058
USCH-37d		21		6	73	0.00486	84	16	0.00072
USCH-83d		17		5	78	0.00458	79	21	0.00073
USCH-180d		25		12	63	0.00590	72	28	0.00108
USCH-C-180d		71			29	0.00592	–b	–	–
UJA-9d	100					0.00106	99	1	0.00034
UJA-22d	100					0.00212	99	1	0.00080
UJA-58d	100					0.00082	99	1	0.00046
UJA-144d	100					0.00168	99	1	0.00014
UJA-180d	100					0.00518	98	2	0.00164
UJA-C-180d	100					0.00083	–	–	–

a The component sums were normalized to 100% (initial range was 94–103% for Fe LCF-EXAFS and 100–102% for U LCF-XANES).

b (−) no data.

c R-factor = Σ((data-fit)^2^/Σdata^2^).

In contrast, the incubated UJA samples underwent no or indiscernible mineralogical/structural transformation over the 180 days, as their XRD and Fe EXAFS spectra were almost identical with the spectrum of the initial UJA (Figures S1b, S2b) and the LCF-EXAFS analysis predicted jarosite only (Table 1). Similarly, the SEM-EDS analyses did not reveal any noticeable morphological/mineralogical alterations of the UJA grains over the 180 days (Figures S3–S4). Instead, the mineral grains (∼1–4 μm in diameter) remained largely intact, retaining regular near-spherical shapes and smooth surfaces as well as stable Fe:S:K ratios that are typical for jarosite (Figures S3–S5 and Table S4).

### 3.4 Solid-Phase Stability and Evolution of U

The U L_3_-edge XANES spectra and their first derivatives of the USCH incubation revealed a partial and progressive reduction of U. This was evidenced by a small but gradual downward shift in the energies of the absorption edge and peak, along with a minor attenuation of the uranyl shoulder feature (∼17190 eV) from day 16 onward (Figure 1). In accordance, the LCF analysis predicted that the fraction of U(IV) overall increased over time and accounted for 28% of the total U at the end of the incubation (Table 1). In comparison, the XANES spectra and their first derivatives of the incubated UJA samples overlapped with those of the initial UJA (Figure 1). This pointed to the persistence of U(VI) throughout the experiment, supported by the results of the U LCF-XANES (Table 1).

The U L_3_ edge EXAFS spectra of the solid phases of the USCH incubation remained largely unchanged on days 9 and 12 (Figure 2a) and thus, were well-reproduced by the same structural model as for the USCH (Figure 2b-c; Table S1). However, by day 16 and onward the oscillations between 4 Å^–1^ and 10 Å^–1^ were somewhat suppressed (Figure 2a), reflecting decreased contributions from the U–O_ax_ scattering path due to the reduction of U(VI),^57^ and the FT peak corresponding to U–Fe path at ∼3.1 Å (uncorrected for phase shift) was strongly suppressed and cannot be fitted with reasonable parameters. Instead, a small but pronounced FT peak appeared at ∼3.5 Å (uncorrected for phase shift) and was best fitted with U–P and U–O_eq_-P scattering paths with a U–P distance of ∼3.9 Å (Figure 2b-c; Table S1). Attempts at fitting the FT peak using U atoms did not converge or gave a negative coordination number. In comparison, the U L_3_-edge EXAFS spectra of the UJA samples remained largely unchanged throughout the incubation (Figure 2d) and accordingly, were well-fitted with a structural model similar as for the initial UJA (Figure 2e-f; Table S1).

### 3.5 Microbial Community Development

16S rRNA gene amplicon sequencing produced a total of 19,674,190 sequences with a mean of 317,325 (min-max: 10–898,130) sequences from 59 samples. Quality control and filtering preserved a total of 19,464,542 and 12,431,901 sequences, respectively (Table S5). Rarefaction curves showed that the number of unique amplicon sequence variants (ASVs) in the individual samples were predominantly asymptotic, indicating that most of the bacterial diversity had been identified (Figure S10). Shannon’s diversity, richness, and Pielou’s evenness data are presented in Figure S11 and described in Text S4.

**Figure 4:**
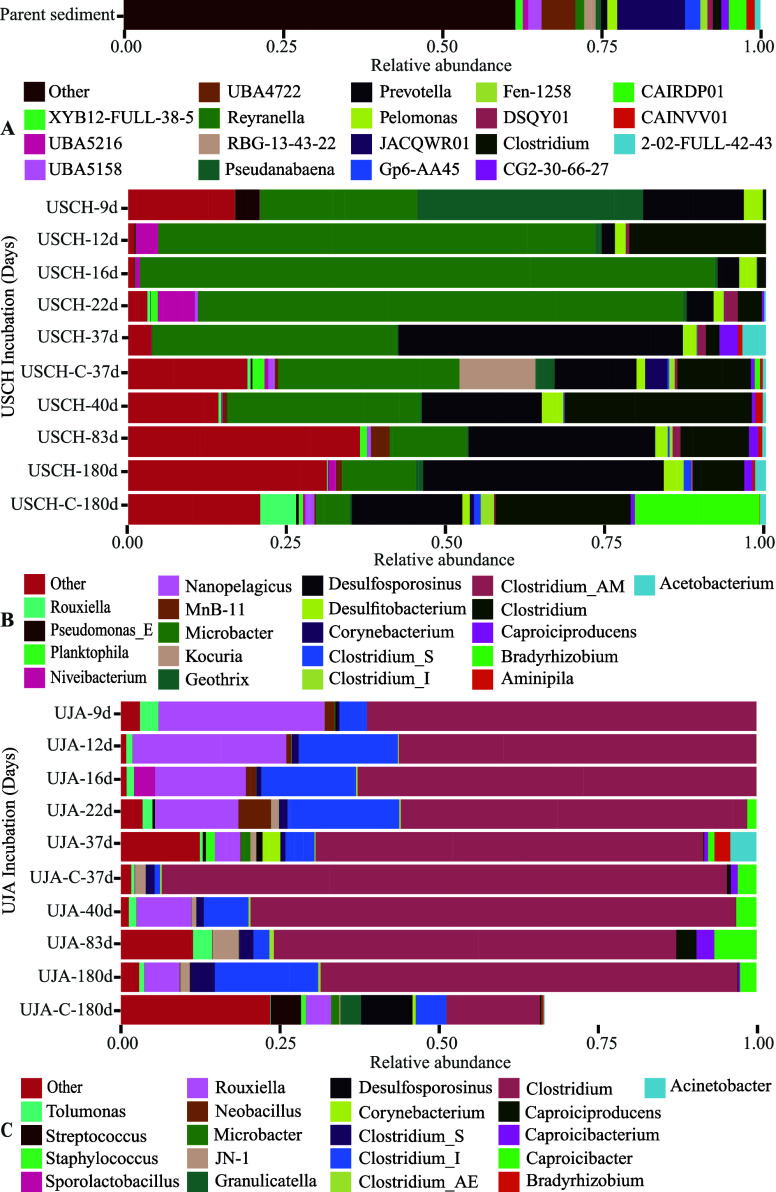
16S rRNA gene amplicon-based composition of the indigenous microbial community (at the level of genera) in the parent sediment (A) and its temporal evolution over the course of the incubation experiments with U(VI)-sorbed schwertmannite (B) and U(VI)-sorbed jarosite (C), respectively, in comparison to the abiotic controls. Missing samples were due to contamination. Samples represent averages of biological replicates (*n* = 3) except for the 180-day control from the U(VI)-sorbed jarosite where the bar covers 0.66 of the relative abundance (*n* = 2).

The relative abundances of the microbial communities were presented at the level of genera with the “other” grouping being consistent throughout the higher taxonomic levels (Figure S12). The sediment used for inoculating the USCH and UJA samples was inhabited by a microbial community dominated by the dissimilatory sulfate reducing genus *JACQWR01* (Desulfobacterota; Figure 4a).^58^ The mean ASV counts for the USCH incubation experiment showed a general increasing trend while the control samples maintained a low ASV count over the incubation experiment (one replicate of the 180-day control was removed due to contamination; Table S5). The genera with the highest relative abundances (Figure 4b) on day 9 included ASVs most similar to *Geothrix* (35.5%), which can use large organic acids as electron donors, Fe^3+^ as an electron acceptor, and releases acetate and succinate.^59^ Further genera included *Microbacter* (25.6%) that is cultured on glucose and yeast extract with the primary fermentation products being lactate and acetate.^60^ This was followed by *Desulfosporosinus* (15.6%) that can couple lactate oxidation to sulfate and thiosulfate reduction;^61^*Pesudomonas* (3.8%) previously isolated from uranium mine waste;^62^ and *Desulfitobacterium* (3.0%) that uses lactate as the electron donor, with Fe^3+^, citrate, or elemental sulfur as electron acceptors^63^ and may also support U reduction.^64^ By day 12, the genera with the highest relative abundances were *Microbacter* (68.6%), *Clostridium* (22.0%), *Niveibacterium* (3.5%), *Desulfosporosinus* (2.1%), and *Desulfitobacterium* (1.7%). In addition, *Microbacter* further dominated the community on day 16 (90.2%) along with *Desulfitobacterium* (2.8%) and *Desulfosporosinus* (3.3%) that both increased, while *Clostridium* (1.5%) strongly decreased in relative abundance. Throughout the remainder of the experiment, relative abundances of *Microbacter* steadily declined; *Desulfitobacterium* fluctuated between 1.6% (day 22) and 3.4% (day 180); and *Desulfosporosinus* spiked on day 22 and then also fluctuated.

The dominant ASVs identified in the UJA incubation (Figure 4c) were consistent with glucose fermentation and yeast extract consumption from the growth media as shown by the stable relative abundances of *Clostridium*,^65^*Riouxella*,^66^ and *Tolumonas*.^67^ Any ferric reduction was likely performed by an unidentified minor community component or was part of the “Other” portion of the stacked bars. The mean ASV counts in the inoculated samples, which were approximately 40-fold higher than the controls, remained stable throughout the incubation experiment (Table S5).

## 4 Discussion

### 4.1 Divergent Reductive Transformation of Schwertmannite and Jarosite: An Interplay of Microbial Activity and Mineral Stability

The USCH samples underwent intense and progressive reductive dissolution (Figure 3 and Table S2). This was accompanied by a rapid and extensive solid-phase transformation to goethite (with ferrihydrite being a possible intermediate phase) along with Fe-monosulfides (Table 1 and Figures S1, S2, S6–S8). These processes likely proceeded through two consecutive stages closely linked to the development of the microbial communities. During the first stage (days 1–22), ASVs that aligned with *Geothrix* and *Microbacter* dominated the microbial communities and the relative abundance of the latter strongly increased from day 9 to 22 (Figure 4b). These two populations likely cooperated whereby *Microbacter* fermented the glucose to lactate and acetate,^60^ while the *Geothrix* used lactate coupled to Fe(III) as an electron acceptor^59^ that consumes acidity during the reduction of schwertmannite-bound Fe(III).^12,22^ Thus, the metabolic activity of these populations was likely the primary biological driver for the rapid buildup of Fe(II)_(aq)_ and the steady increase in pH during the first stage (Figure 3a,c). These processes in turn created favorable conditions (e.g., high levels of Fe(II)_(aq)_ and circumneutral pH conditions) for the adsorption of Fe(II)_(aq)_ onto schwertmannite surfaces and subsequent electron transfer to structural Fe(III), driving rapid dissolution and transformation of schwertmannite to goethite^14,69^ as observed from day 12 (Table 1). The transition of acidic to circumneutral pH and accumulation of lactate/acetate during stage 1 were also beneficial for and potentially assisted the growth of sulfate reducers,^24,59^ which prefer low-molecular-weight organics (e.g., lactate and acetate) and relatively high pH.^70^ From day 37 (stage 2), the relative abundance of ASVs most similar to *Desulfosporosinus* (a genus of sulfate reducers using lactate as an electron donor^61^) strongly increased and dominated the microbial communities. The generation of sulfide by populations related to *Desulfosporosinus* was potentially responsible for the removal of Fe(II)_(aq)_ and S_(aq)_ (Figure 3b,c) via the observed precipitation and accumulation of black Fe-monosulfides (as verified by the LCF-EXAFS analysis and SEM-EDS observations). During this incubation stage (37–180 days), the decreasing availability of glucose along with the rise in pH above *Microbacter*’s optimal growth range (pH 6.5^60^) were the likely reasons for the decline in the relative abundance of this population. In contrast, a low relative abundance of *Clostridium* was maintained until day 180. Thus, these populations likely continued metabolizing the remaining glucose and yeast extract, fueling *Desulfosporosinus* to reduce sulfate. These results highlight the high capability of natural microbial communities to evolve and drive a rapid and successive reduction of Fe(III) and sulfate bound to schwertmannite, as observed for cocultured iron(III)- and sulfate-reducing bacteria.^71,72^

Despite being inoculated and incubated under similar biogeochemical conditions as the USCH, the UJA samples exhibited neither observable reductive dissolution (as reflected by the fairly stable concentrations of K_(aq)_, Table S3) and morphological/mineralogical alterations nor pH increase over time (Tables 1, S3, and Figures 3g, S1–S4). Accordingly, the microbial communities in these samples remained largely unchanged during the incubation (Figure 4c) and were dominated by ASVs most similar to the fermentative *Clostridium*, *Riouxella*, and *Tolumonas* genera that metabolize glucose and yeast extract. Although surface area has been proposed to be a primary control on the rates of microbial Fe(III) oxyhydroxide reduction,^73,74^ the specific surface area of the UJA was only approximately half that of the USCH and thus, was unlikely the main cause for its slow/limited reductive dissolution and transformation. However, the UJA was much more crystalline than the USCH, as evidenced by its intense and well-defined diffraction peaks in contrast to the broad and poorly visible peaks of the USCH (Figure S1). It has also been proposed that the solubility of Fe(III) hydroxides (reflecting thermodynamic stability of the Fe(III) atoms on Fe(III)-hydroxide surfaces) is a good predictor for maximum cell-specific Fe reduction rates of a range of Fe(III) hydroxides, thus exerting a strong impact on the rate and extent of microbial Fe(III)-hydroxide reduction.^75,76^ In line with the low crystallinity of the USCH, schwertmannite is reported to have much higher solubility products (log *K*_sp_ = 7.1–18.0^77−79^) than jarosite (log *K*_sp_=-7.12– −14.56^80^). Taken together, the divergent reductive dissolution and associated transformation of the USCH and UJA samples was most probably caused by the substantial difference in their crystallinity, governing the thermodynamic stability and bioaccessibility/reducibility of Fe(III) on their surfaces.

While electron transfer between surface-sorbed Fe(II) and structural Fe(III) can trigger rapid transformation of jarosite to Fe hydroxides of higher stabilities,^36,37,81^ low pH conditions (e.g., pH = 4) can effectively retard the transformation by blocking the surface adsorption of Fe(II)_aq_, even at concentrations as high as 20 mM Fe(II)_aq_.^37^ Over the UJA incubation experiment, the pH of the solutions (3.8–4.7) was well below the pH_pzc_ (5.6–7.8) reported for jarosite,^26,82^ meaning that the incubated UJA surfaces remained positively charged and was thus electrostatically unfavorable for Fe(II)_aq_ adsorption. Consequently, the low-pH conditions, in combination with the high crystallinity of the UJA, effectively inhibited Fe(II)-mediated transformation of these samples during the incubation.

### 4.2 Controls on the Stability and Repartitioning of Surface-Sorbed U

Despite being exposed to approximately 1–2 mM Fe(II)_(aq)_, the ternary ≡FeO_surface_-U(VI)-carbonate/bicarbonate complexes loaded on the UJA samples remained chemically inert during the incubation (Tables 1, S1 and Figure 3). This was consistent with (i) the high mineralogical and chemical integrity of the incubated UJA grains (Table 1 and Figures S1–S4) and (ii) the low-pH conditions (pH = 3.8–4.7, Figure 3g) that not only lowered the thermodynamic potential for electron transfer between Fe(II)_(aq)_ and U(VI)_(aq)_,^83^ but also disfavored Fe(II)_(aq)_ binding to the surface-sorbed U(VI)-carbonato complexes and any associated surface-catalyzed electron transfer.^84,85^ This is consistent with the facts that (i) previous experiments where Fe(II)-mediated U reduction was observed were conducted at much higher (circum-neutral) pH^85−90^ and (ii) abiotic U reduction in U(VI)- and Fe(II)-spiked natural sediments (pH = 7.4 and 8.3) only occurred in the presence of sufficient amounts of surface-sorbed Fe(II), especially oligomeric Fe(II) species.^91^

Similarly, the low-pH conditions and limited mineralogical alteration of the USCH grains by day 12 likely facilitated the persistence of the ternary ≡FeO_surface_-U(VI)-carbonate/bicarbonate complexes during the early stage of incubation (Figures 2–3, S1–S2, S5–S6 and Tables 1, S1). Given the well-defined role of surface-sorbed Fe(II) in catalyzing the reductive transformation of schwertmannite to goethite,^12,14,22,23,69^ the formation of goethite between day 12 and 16 provided indirect evidence for the strong adsorption of Fe(II)_(aq)_ onto the USCH grains during this period. This was probably favored by the very high levels of Fe(II)_aq_ at weakly acidic pH (Figure 3a,c). Under inorganic/abiotic and circum-neutral pH conditions, Fe(II)-catalyzed recrystallization of Fe oxyhydroxides in the presence of U(VI) commonly led to the formation of U(V)-incorporated goethite.^87−89,92^ This is because the reduction of U(VI) to U(V) (after Fe(II)_(aq)_ binds to surface-sorbed U(VI)) can overcome a mismatch in coordination of U(VI) with the Fe sites in the goethite structure.^92^ However, the U EXAFS data of the USCH samples after day 16 displayed no discernible U–Fe atomic shell. This suggested that U was neither sorbed via surface Fe sites on remaining schwertmannite or neo-formed goethite^93^ and Fe-monosulfides,^94^ nor structurally incorporated into neo-formed goethite.^87−89,92^ Instead, the U in these samples was, on average, coordinated to 1–2 C and 0.3–0.5 P at ∼2.9 Å and ∼3.9 Å, respectively (Figure 3 and Table S1). Although the U–P distance was slightly longer than those reported for typical monodentate binding of U(IV/VI) to phosphate ligands (∼3.6–3.8 Å),^95−103^ it was smaller than the sum of the U–O_eq_ distances (∼2.4 Å, Table S1) plus P–O distances of a phosphate ligand (∼1.6 Å)^104^ and thus, still chemically feasible. These data and features collectively implied that when the USCH was being redissolved and transformed to goethite, its surface-sorbed U was liberated, partially reduced, and eventually complexed with carbonyl and phosphoryl ligands (on organic substances or biomass) as monomeric U(IV)/ U(VI) species. The complexation of U with phosphoryl ligands might have been partially contributed by increased production of phosphate-rich extracellular polymeric substances due to a toxicity response of microbial cells to the high levels of U in the incubation system.^98,102^ The formation of monomeric U(IV) complexes, which could effectively inhibit the formation of uraninite,^98,100,101,103,105^ also explained the absence of a uraninite phase (as indicated by the lack of strong U–U FT peaks typical of uraninite, Figure 2b) during the partial reduction of U on the USCH samples.

### 4.3 Environmental Implications

The results of this study are environmentally relevant, given that schwertmannite and jarosite are widespread in many acidic and sulfate-rich environments and can adsorb high levels of U as shown here and previously.^19^ In particular, materials containing these minerals might be more frequently flooded in the future as a result of climate change, land-use change or remediation activities, thus potentially releasing U and other metals due to reductive dissolution of the oxyhydroxysulfates. In this study new insights into these processes were provided. First, U(VI)-sorbed schwertmannite stimulated and sustained intensive metabolic activities of iron- and sulfate- reducing bacterial communities (Figure 4b), thereby acting as a driver for acidity consumption and Fe(II)_aq_/sulfide production. Second, the reductive transformation of U(VI)-sorbed schwertmannite did not increase the risk of U reliberation into solution as dissolved species (Figure 3e–f). This was due to the formation of monomeric U(VI)/U(IV) complexes with carboxyl and phosphoryl ligands of biomass and organic substances (Tables 1, S1; Figure 2a–c). These results thus highlighted the important roles of biomass (e.g., extracellular polymeric substances) and organic substances in attenuating U, especially considering that they effectively inhibited (i) the formation of highly soluble Ca-carbonato-U(VI) complexes that were favored by the high levels of dissolved Ca and alkaline pH conditions toward the end of the experiment (Figure 3; Table S2) and (ii) the nucleation of uraninite during the partial reduction of U(VI). Third, the data revealed that inner-sphere U complexes adsorbed on jarosite had a high chemical resistance toward evolving solution conditions, as they remained chemically inert throughout the incubation experiment (Figures 1, 2; Tables 1, S1). This was noteworthy, especially considering that that the UJA samples underwent limited reductive dissolution (Figure 3h, i), experienced a moderate change in pH conditions (Figure 3g), and contained abundant strong U-complexing ions/agents (e.g., phosphate, organic acids, and humic acid derived from the initial artificial groundwater and biomass/organics produced by microbial activities). Lastly, our data suggested that the indigenous microbial community had low ability to trigger a notable reductive transformation of the U(VI)-sorbed jarosite under acidic conditions (Table 1; Figure 3g–i). However, the development of alkaline pH conditions and production of Fe(II)_aq,_ and sulfides by microbial mediated reduction of co-occurring schwertmannite (and/or other poorly crystalline Fe(III) oxyhydroxides), as shown here (Figure 3a–c, S8; Table 1) and previously,^12,22−24,106^ might favor initiating and maintaining reductive transformation also of jarosite in the natural environment.

## Data Availability

16S rRNA gene amplicon sequences are available in the European Nucleotide Archive (ENA) at EMBL-EBI (https://EBI.ac.uk/ena) under the project accession number: PRJEB72686 and sample accession numbers: ERS18329019 through ERS18329080. Microbial data analysis and figures were prepared in R and the full reproducible code is available in Supporting Information Text S3.

## Supporting Information Available

Detailed information on the preparation of U(VI)-sorbed jarosite and schwertmannite, XAS data analysis, microbial analyses, results of microbial diversity indices plus additional figures and tables showing XRD spectra, Fe EXAFS spectra, SEM images and EDS mapping results, rarefactions curves of the ASV counts versus the sequencing depth, changes in microbial diversity indices and microbial community composition at different levels, U EXAFS fitting results, changes in water chemistry, SEM-EDS results of selected points, details of DNA sampling, Illumina sequencing, and data processing, results of one-way ANOVA and Tukey’s posthoc tests (PDF)
