# Supplementary material for: Uranium Repartitioning during Microbial Driven Reductive Transformation of U(VI)-Sorbed Schwertmannite and Jarosite
Source: Environ Sci Technol. 2024 Oct 3;58(41):18324–34. doi: 10.1021/acs.est.4c03645 (PMC11483811; doi:10.1021/acs.est.4c03645)
Supplement: Supplementary file 1 — es4c03645_si_001.pdf [file es4c03645_si_001.pdf]

Supporting information (SI) for:

Uranium repartitioning during microbial driven reductive transformation of U(VI)-sorbed schwertmannite and jarosite

Changxun Yu[a*], Anders Johnson[b], Andreas Karlsson[c], Roman Chernikov[d], Viktor Sjöberg[e], Zhaoliang Song[f], Mark Dopson[b], Mats E. Åström[a]

[a]*Department of Biology and Environmental Science, Linnaeus University, 39231 Kalmar, Sweden*

[b]*Centre for Ecology and Evolution in Microbial Model Systems (EEMiS), Linnaeus University, 39231 Kalmar, Sweden*

[c]*Department of Geosciences, Swedish Museum of Natural History, 10405 Stockholm, Sweden*

[d]*Canadian Light Source, 44 Innovation Boulevard, Saskatoon, SK S7N 2V3, Canada*

[e]*Man-Technology-Environment Research Centre (MTM), Örebro University, 70182 Örebro, Sweden*

[f]*Institute of Surface-Earth System Science, School of Earth System Science, Tianjin University, 300072, Tianjin, China*

[*] Corresponding author

Email address: changxun.yu@lnu.se; yuchangxun2006@163.com

Number of pages (38)

Number of Figures (12)

Number of tables (13)

Text S1: Detailed procedures for the preparation of U(VI)-sorbed jarosite and schwertmannite.

The U(VI)-sorbed jarosite (UJA) and schwertmannite (USCH) for the incubation experiments were prepared as follows. Jarosite and schwertmannite were synthesized following Baron and Palmer[1] and Regenspurg et al[2], respectively. The resulting precipitates were repeatedly washed with ultrapure water and then resuspended in 2 L ultrapure water. The pH of the suspensions was adjusted to 4.5 with 0.5 M KOH. When the pH stabilized, 5 mL 85 mM U stock solution was added incrementally to each of the two suspensions within 2 h. The suspensions were vigorously stirred for 12 h, during which the pH was regularly readjusted. Thereafter, the mineral particles were obtained via centrifugation (8000 g for 10 min) and repeatedly washed with ultrapure water, before being oven-dried at 40 ℃ overnight. The dry minerals were finely pulverized using an agate mortar.

Text S2: Details of linear combination fitting of the Fe EXAFS and U XANES spectra plus shell-by-shell fitting of the U EXAFS spectra.

The fractional amounts of Fe-bearing minerals in the samples were quantified by linear combination fitting (LCF) using the EXAFS spectrum of the UJA or the USCH, together with the EXAFS spectra of goethite (Sigma-Aldrich) and magnetite (Sigma-Aldrich) as well as synthetic 2-line ferrihydrite[3] and pyrite[4] that were collected in parallel with the samples using the same experimental set-up plus the spectra of siderite and FeS reported previously.[5, 6] The LCF analysis was started with the two references giving the lowest R-factor and additional reference spectra were included stepwise if the R-factor decreased ≥10%. The relative proportions of U(VI) and U(IV) were estimated by LCF of the sample U XANES spectra with the corresponding spectra of uraninite plus UJA or USCH, respectively. The fitted U(VI) and U(IV) fractions depended to some extent on the choice of U(VI) and U(VI) references and are expected to have uncertainties of ±10%.[7]

To gain quantitative structural information about the local environment around the U atoms in the samples, the U EXAFS sample spectra (with *k*-weighting of 1, 2, 3) were fitted simultaneously in R-space (R=1-3.35 to 3.77 Å) using the Artemis program.[8] Theoretical scattering amplitude and phase shifts were calculated with FEFF7 based on the crystal structure of (i) sodium uranyl triacetate[9] (U-O_{ax}, U-O_{eq}, U-C, and three multiple scattering paths of the U-O_{ax}); (ii) schwertmannite[10] or jarosite[11] with U substituted for one Fe (U-Fe); and (iii) triuranyl diphosphate tetrahydrate[12] (U-P and U-O_{eq}-P). For the EXAFS fit, the amplitude reduction factor was set to 0.9, energy shift parameter was fitted as a global parameter for all paths, and the bond distance was floated for each path. For the other paths, the coordination number was floated while the Debye-Waller factor of the U-O_{eq}, U-C, U-Fe, U-P, and U-O_{eq}-P paths was fixed to 0.01 Å^2, 0.004 Å^2, 0.01 Å^2, 0.003 Å^2, and 0.003 Å^2, respectively, as reported by previous studies.[13, 14] These constrains not only reduced the number of variables in the fits, but also facilitated the comparison between the results of the samples.

Text S3: Details of the DNA extraction, 16S rRNA gene PCR amplification, amplicon sequencing, and bioinformatics.

DNA extractions were performed using ~250 mg of solid material from each sample using the Qiagen DNeasy Powersoil kit per the manufacturer's protocol. PCR amplification of the extracted DNA and sequencing library preparation were performed using a two-step technique as previously described.[15] Briefly, the first PCR targeting the V3-V4 16S rRNA gene sequence utilized the 341F and 805R primer pair[16] and the second PCR attached a unique tag for Illumina sequencing to generate the final amplicons (2×300 bp pair-end reads[17]). DNA quantity and quality was investigated by electrophoresis, Qubit 2.0 fluorometer (Thermofisher), and TapeStation (Agilent). The final amplicons were sequenced at the Science for Life Laboratory, Stockholm on the Illumina MiSeq platform as previously described.[15]

Illumina sequencing reads were processed using the Ampliseq pipeline v1.2.[18] This pipeline incorporates QIIME2[19] and DADA2[20] and generated 8,532 unique amplicon sequence variants (ASVs) that were annotated against the SILVA database v138.1[21] as previously described.[22] Downstream processing was performed in R v4.2.1 and RStudio[23] using the packages vegan[24] and tidyverse[25] with the full code available in GitHub (see https://github.com/AndersCJohnson/AndersCJohnson/tree/microbial_reduction_Co-sorption_of_U_with_SCH_and_JA). Due to 16S rRNA gene sequencing data being relative values, the compositional data were transformed for the variance analysis.[26]

Text S4: Temporal evolution of the Shannon's H, species richness, and Pielou's evenness during the two incubation experiments

Shannon's diversity, richness, and Pielou's evenness for the inoculated USCH samples showed a sharp decline from day 9 to day 16, followed by a sharp increase (Figure S11a). The high diversity indices shown in the control samples were likely due to kit contaminants with few ASV counts and low sequencing depth creating artificially high values. An analysis of variance (ANOVA) showed statistically significant differences between Shannon's diversity and days (p<0.001), richness and days (p<0.005), and Pielou's evenness and days (p<0.005) (Table S6). The Tukey's test of multiple means also showed a significant difference in Shannon's diversity, richness, and Pielou's evenness between the USCH samples (Tables S7-9). Through the incubation period, the UJA samples showed a slight increase in Shannon's diversity while the richness and evenness remained mostly stable and there were similar aberrations related to low ASV counts and low sequencing depth as seen with the USCH control samples (Figure S10a,b). The ANOVAs showed that there were significant differences in Shannon's diversity (p<0.05) and evenness (p<0.001) among the UJA samples, but no significant differences in richness (Table S10). The Tukey's test of multiple means showed very few significant differences in Shannon's diversity and evenness among the UJA samples, suggesting very little temporal change with

no significant differences (Tables S11-13). However, there were significant differences between the inoculated samples and the controls, which was expected due to the low ASV counts and/or kit-contaminant noise[27] that would otherwise be muted during cellular growth.

Figure S1. XRD patterns showing changes in mineralogy over the two incubation experiments. S = schwertmannite, J = jarosite, and G = goethite. The d-spacing values of all major diffraction peaks for each of the minerals were given in the brackets.

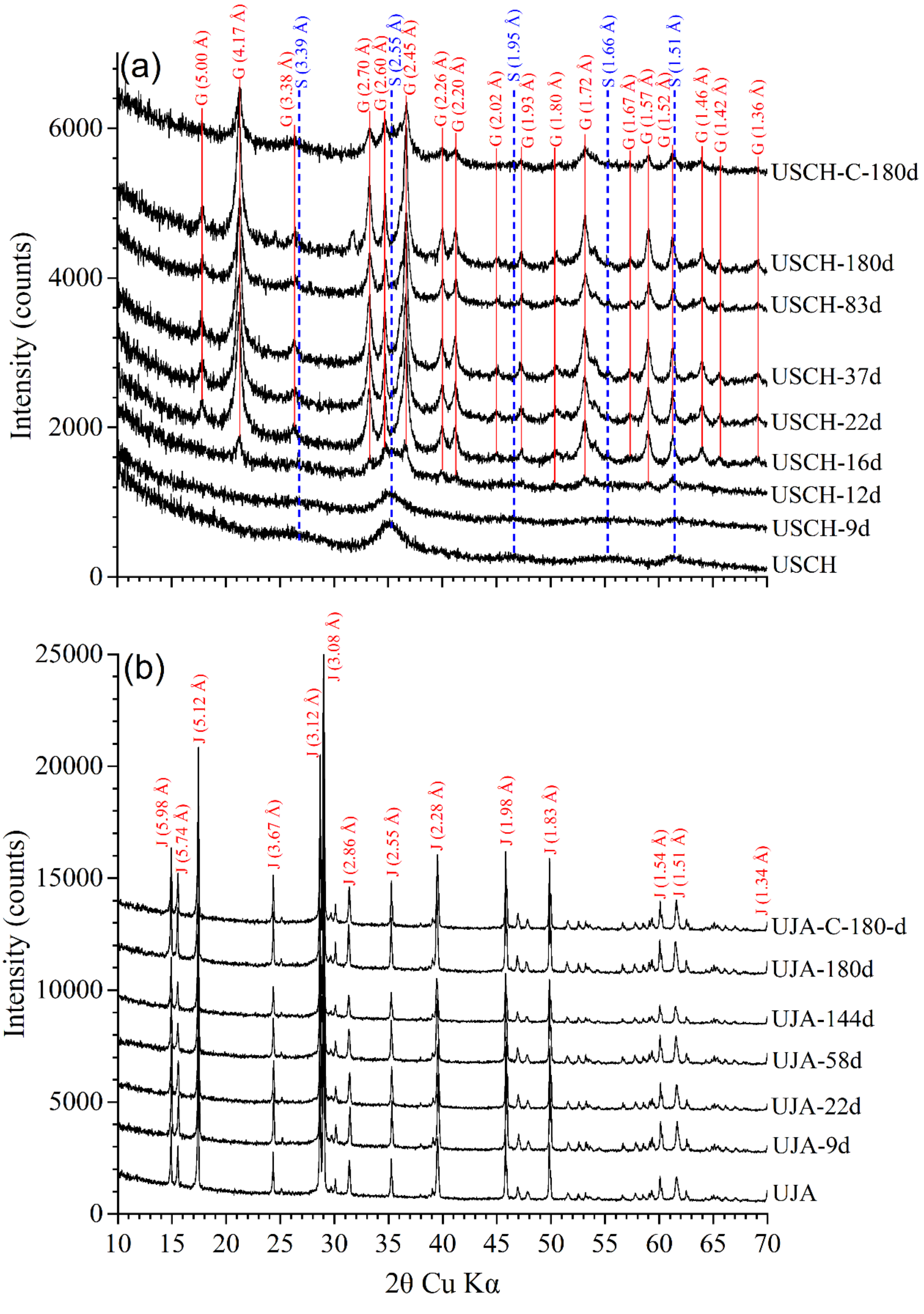

Figure S2. Iron K-edge EXAFS spectra for the solid-phase samples from the two incubation experiments. The dashed lines highlight the distinctive features for the EXAFS spectrum of goethite. USCH and UJA are the initial U(VI)-sorbed schwertmannite and U(VI)-sorbed jarosite, respectively, used for the two incubation experiments.

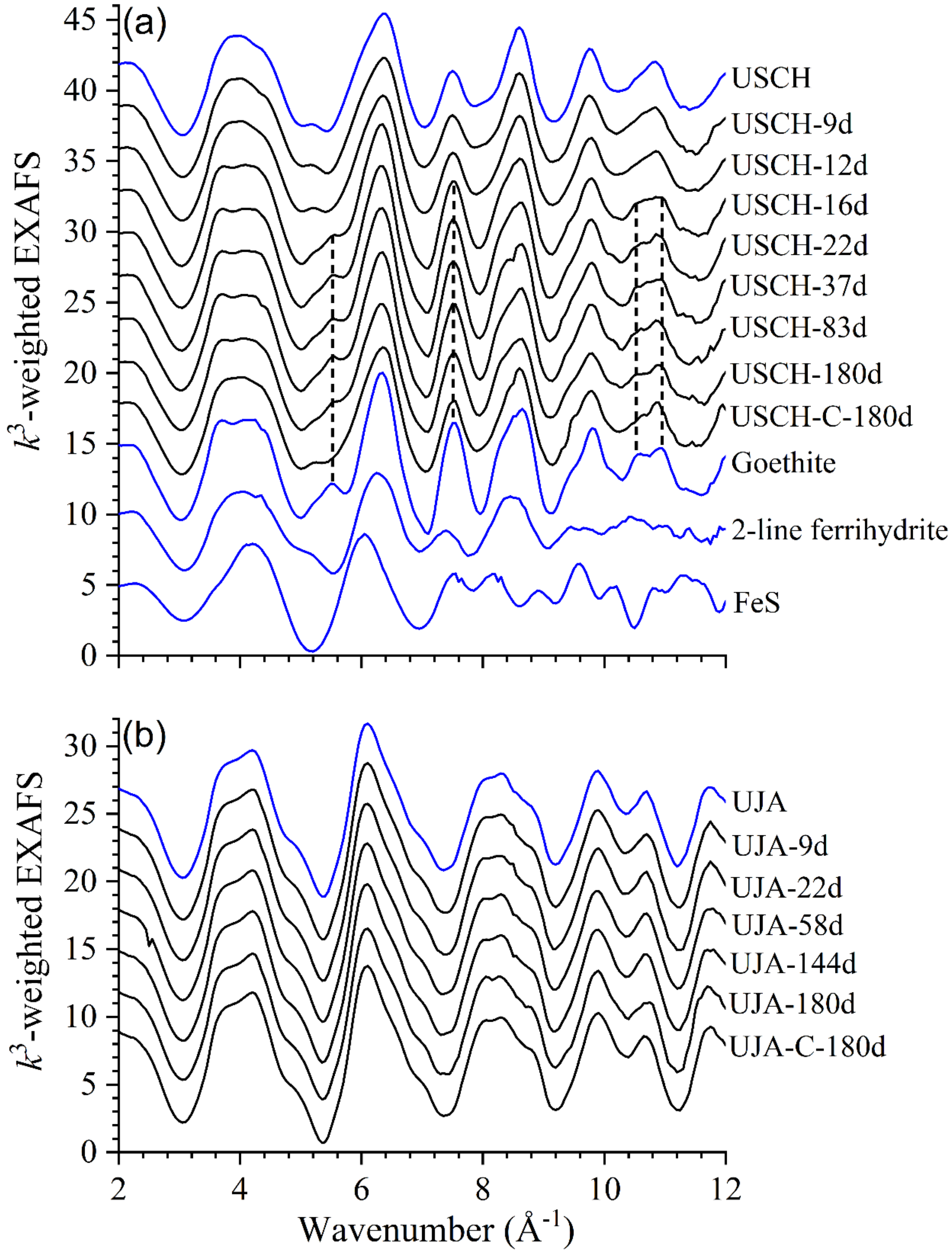

Figure S3. SEM images and EDS mapping results of UJA-9d (triplicate nr 2): (a) low-magnification SEM image, (b) high-magnification SEM image, and (c-g) composite and individual elemental maps of Fe, K, S and O, respectively, in the same subregion as (b). The EDS results for the points marked in (b) are given in Table S4.

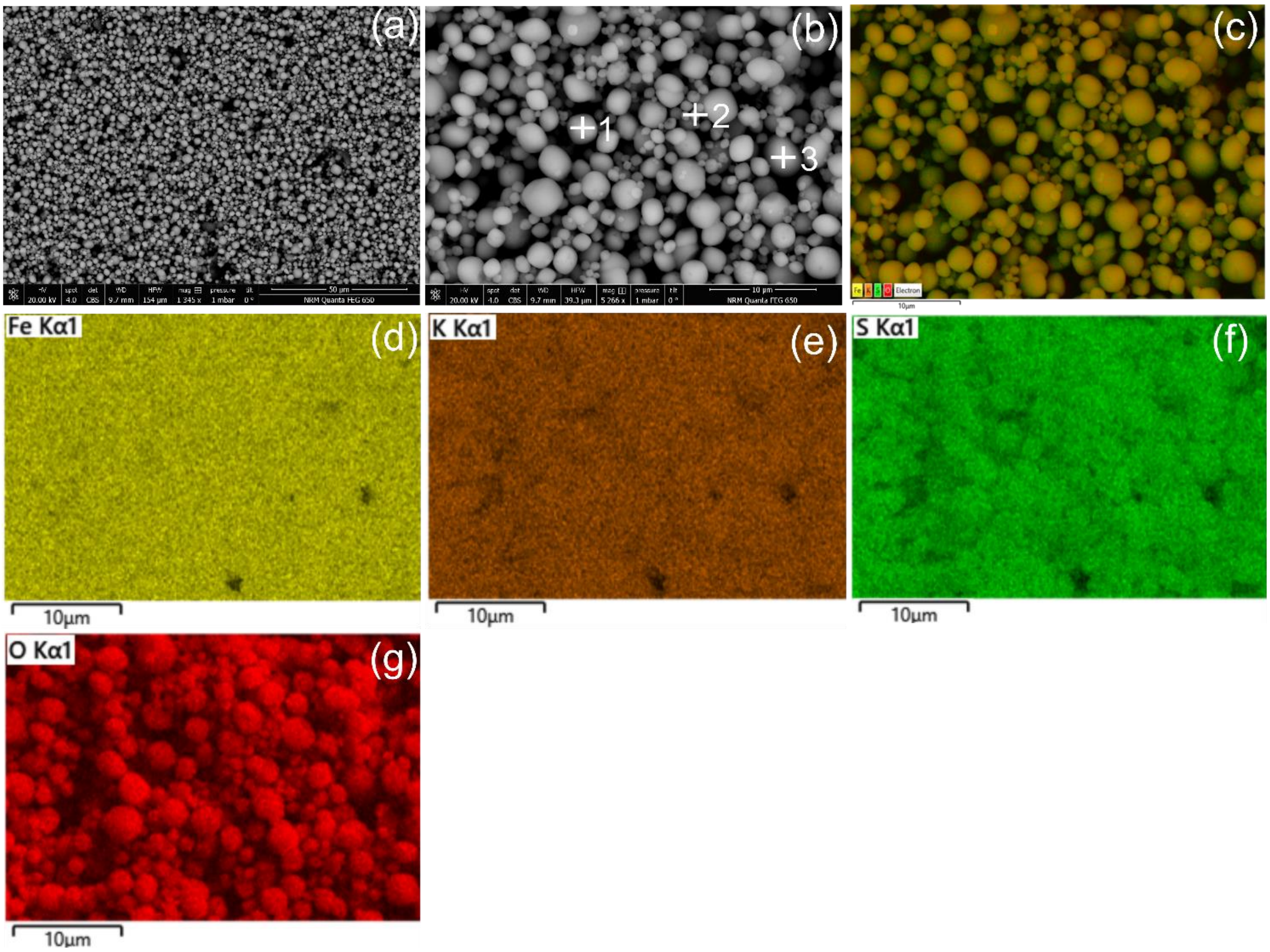

Figure S4. SEM images and EDS mapping results of UJA-180d (triplicate nr 2): (a) low-magnification SEM image, (b) high-magnification SEM image, and (c-g) composite and individual elemental maps of Fe, K, S, and O, respectively, in the same subregion as (b). The EDS results for the points marked in (b) are given in Table S4.

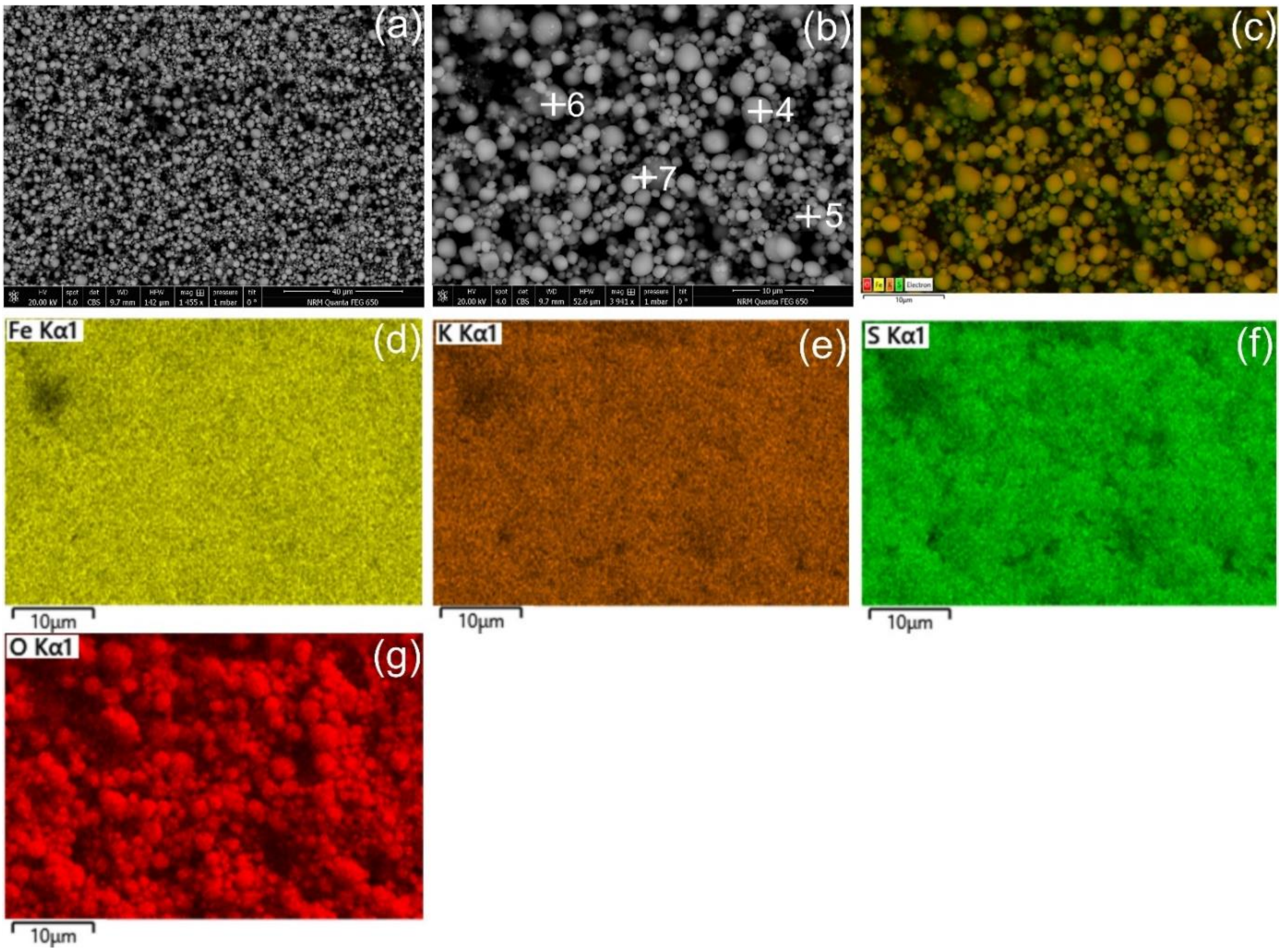

Figure S5. Low (a) and high (b) -magnification SEM images for UJA-C-180d (triplicate nr 2). The EDS results for the points marked in (b) are given in Table S4.

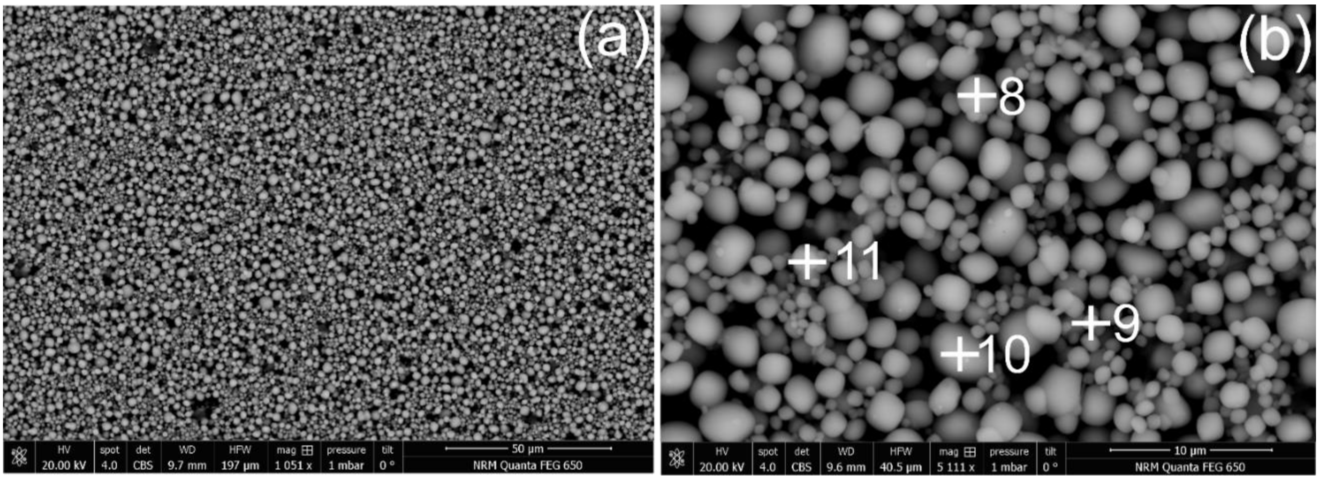

Figure S6. SEM images and EDS mapping results of USCH-9d (triplicate nr 2): (a) low-magnification SEM image, (b) high-magnification SEM image, and (c-i) composite elemental map of Fe, O, and S taken together as well as individual elemental maps of Fe, S, O, Ca, C, and U, respectively in the same subregion as (b). The EDS results for the points marked in (b) are given in Table S4.

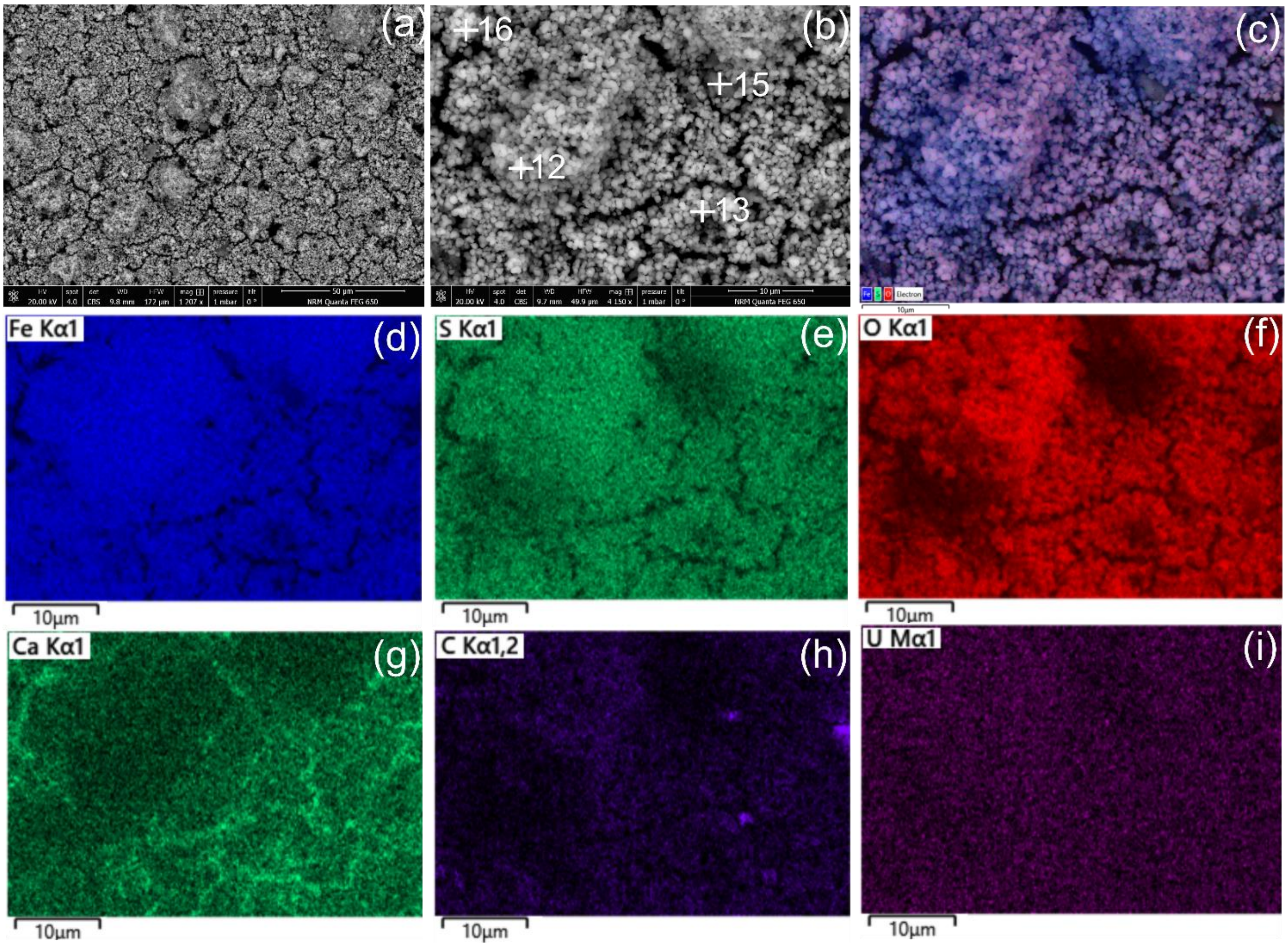

Figure S7. SEM images and EDS mapping results of USCH-12d (triplicate nr 2): (a) low-magnification SEM image, (b) high-magnification SEM image, and (c-i) composite elemental map of Fe, Ca, S, and O taken together as well as individual elemental maps of Fe, O, S, Ca, C, and U, respectively in the same subregion as (b). The EDS results for the points marked in (b) are given in Table S4.

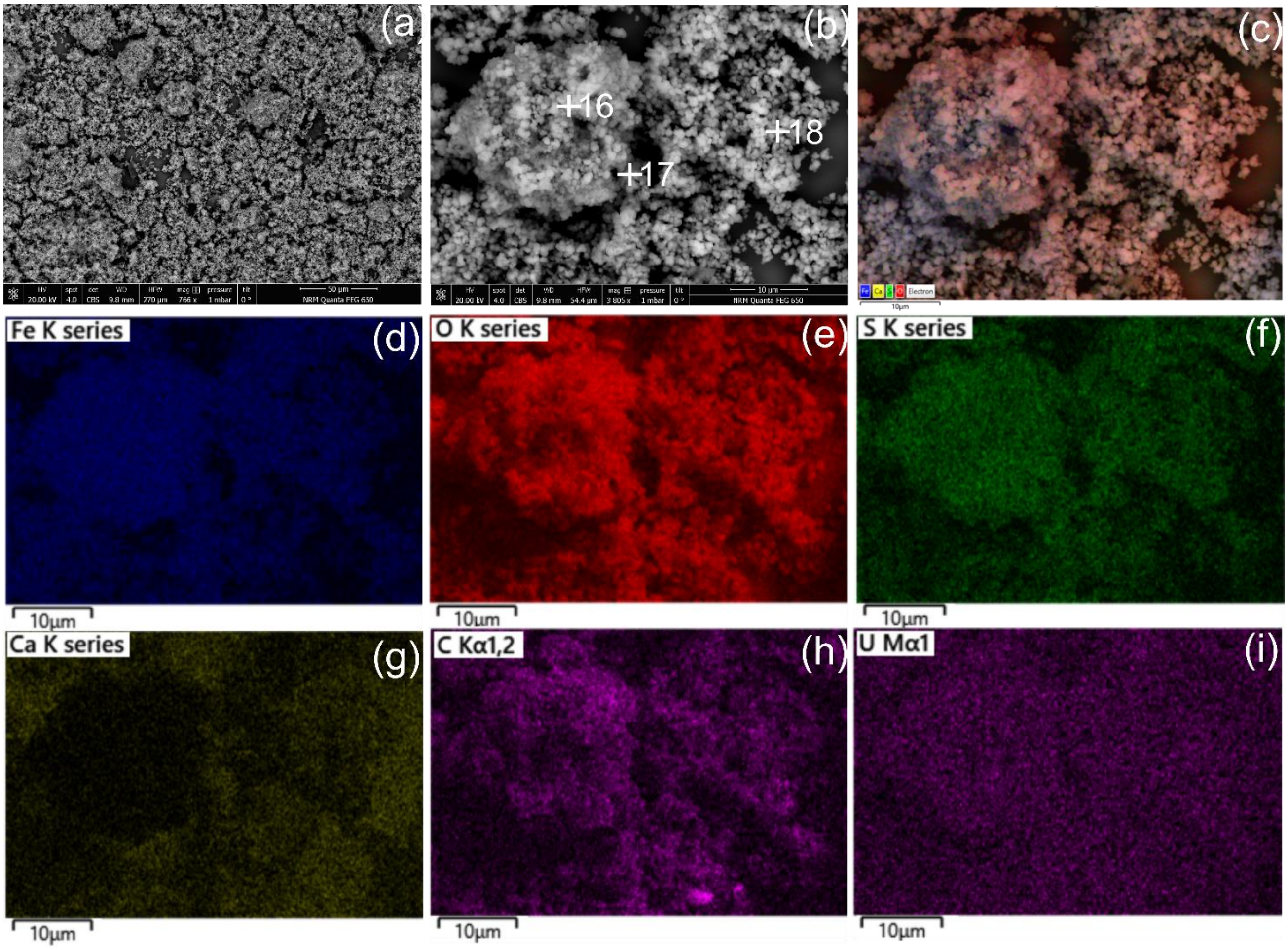

Figure S8. SEM images and EDS mapping results of USCH-180d (triplicate nr 2): (a) low-magnification SEM image, (b) high-magnification SEM image, and (c-i) composite elemental maps of Fe, Ca, S, and O taken together as well as individual elemental maps of Fe, Ca, S, O, C, and U, respectively, in the same subregion as (b). The EDS results for the points marked in (b) are given in Table S4.

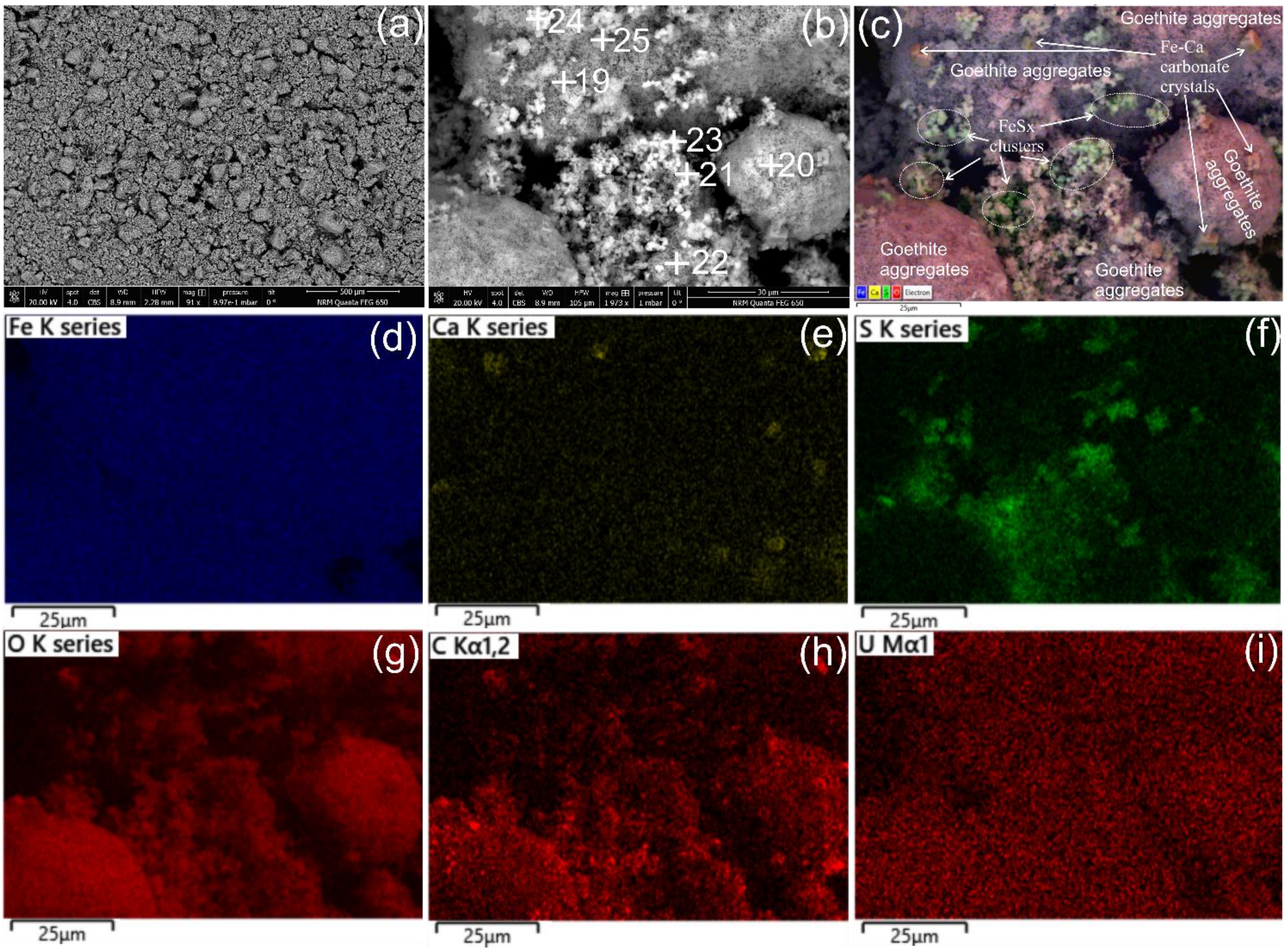

Figure S9. SEM images and EDS mapping results of USCH-C-180d (triplicate nr 2): (a) low-magnification SEM image, (b) high-magnification SEM image, and (c-i) composite elemental maps of Fe, Ca, S, and O taken together as well as individual elemental maps of Fe, S, O, Ca, C, and U, respectively in the same subregion as (b). The EDS results for the points marked in (b) are given in Table S4.

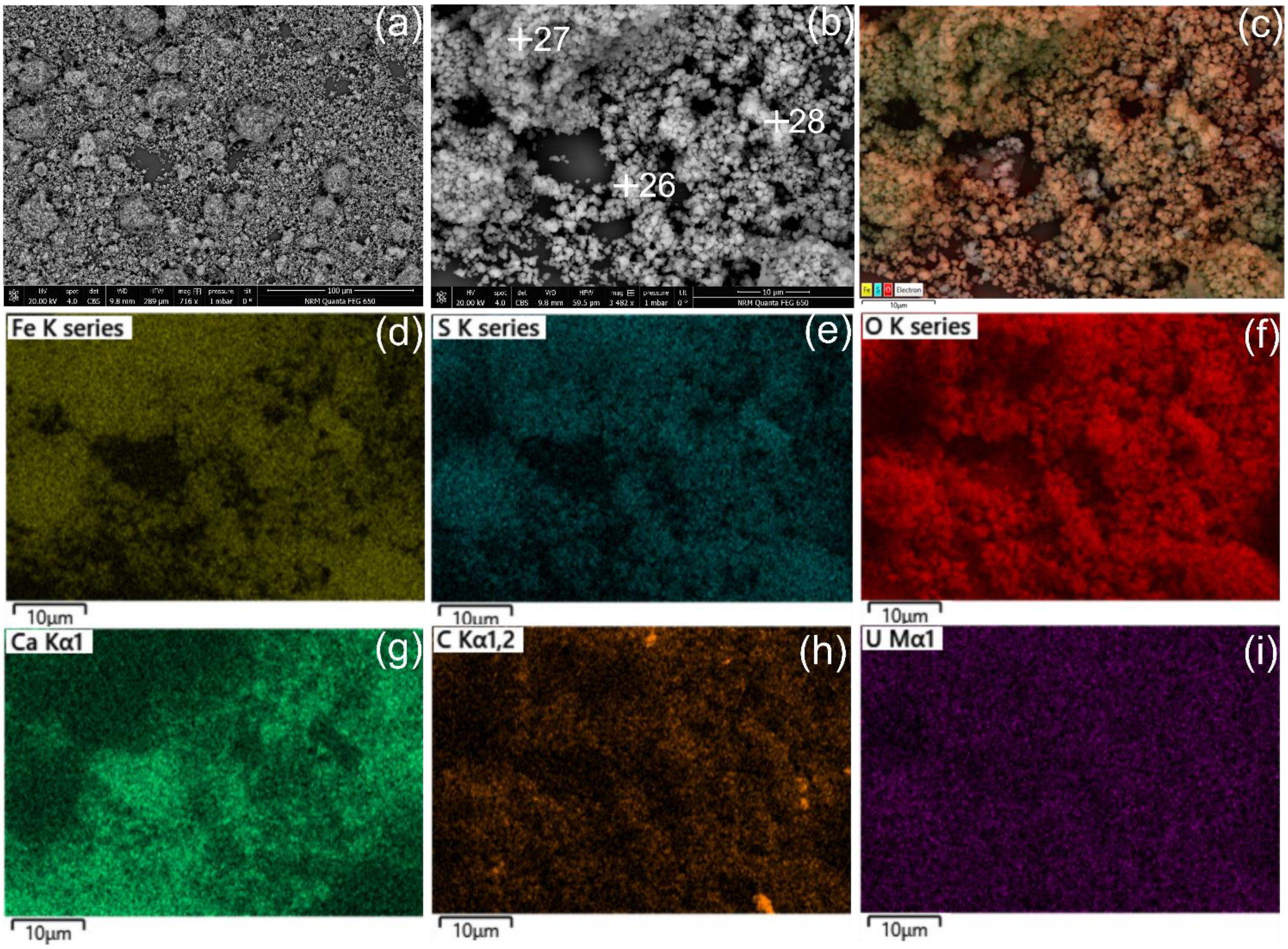

Figure S10. Rarefactions curves showing the ASV counts versus the sequencing depth expressed as the number of reads for the (A) parent sediment inoculum, (B) U(VI)-sorbed schwertmannite, and (c) U(VI)-sorbed jarosite.

(A)

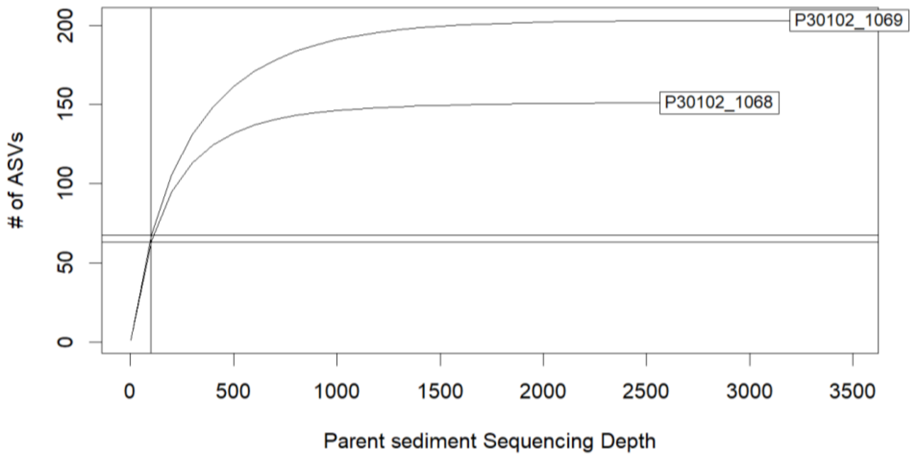

(B)

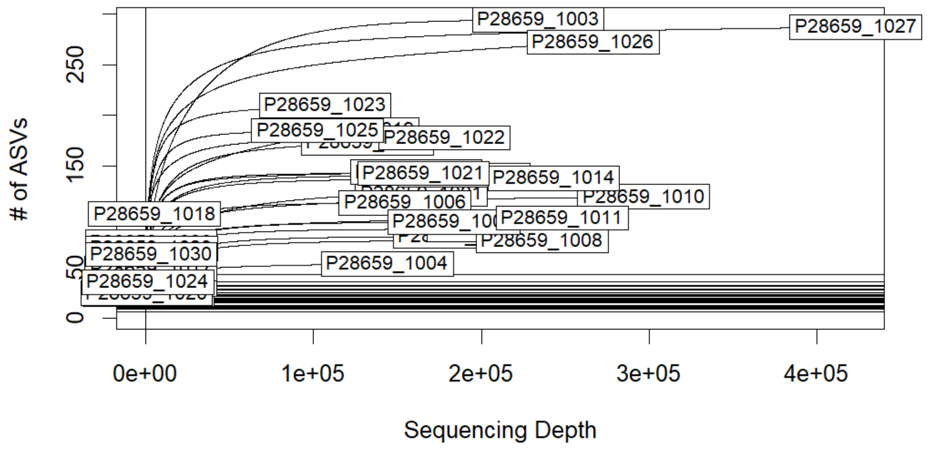

(C)

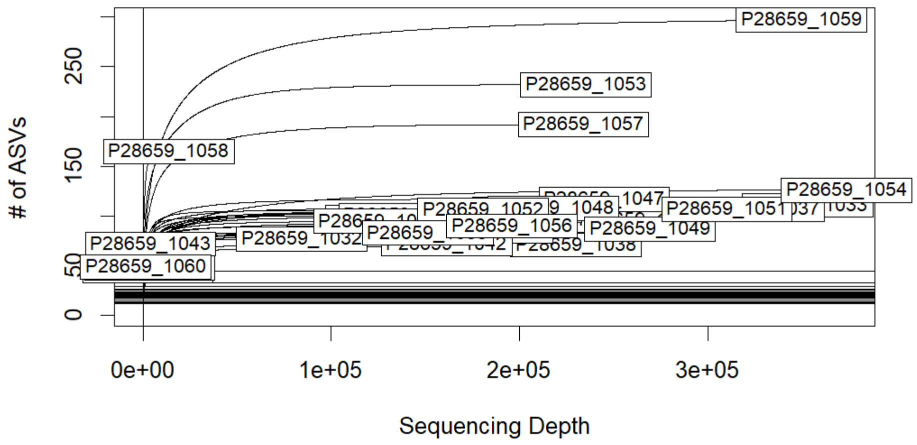

Figure S11. Microbial diversity indices for USCH (A) and UJA_LPH (B) showing Shannon's H, species richness, and Pielou's evenness.

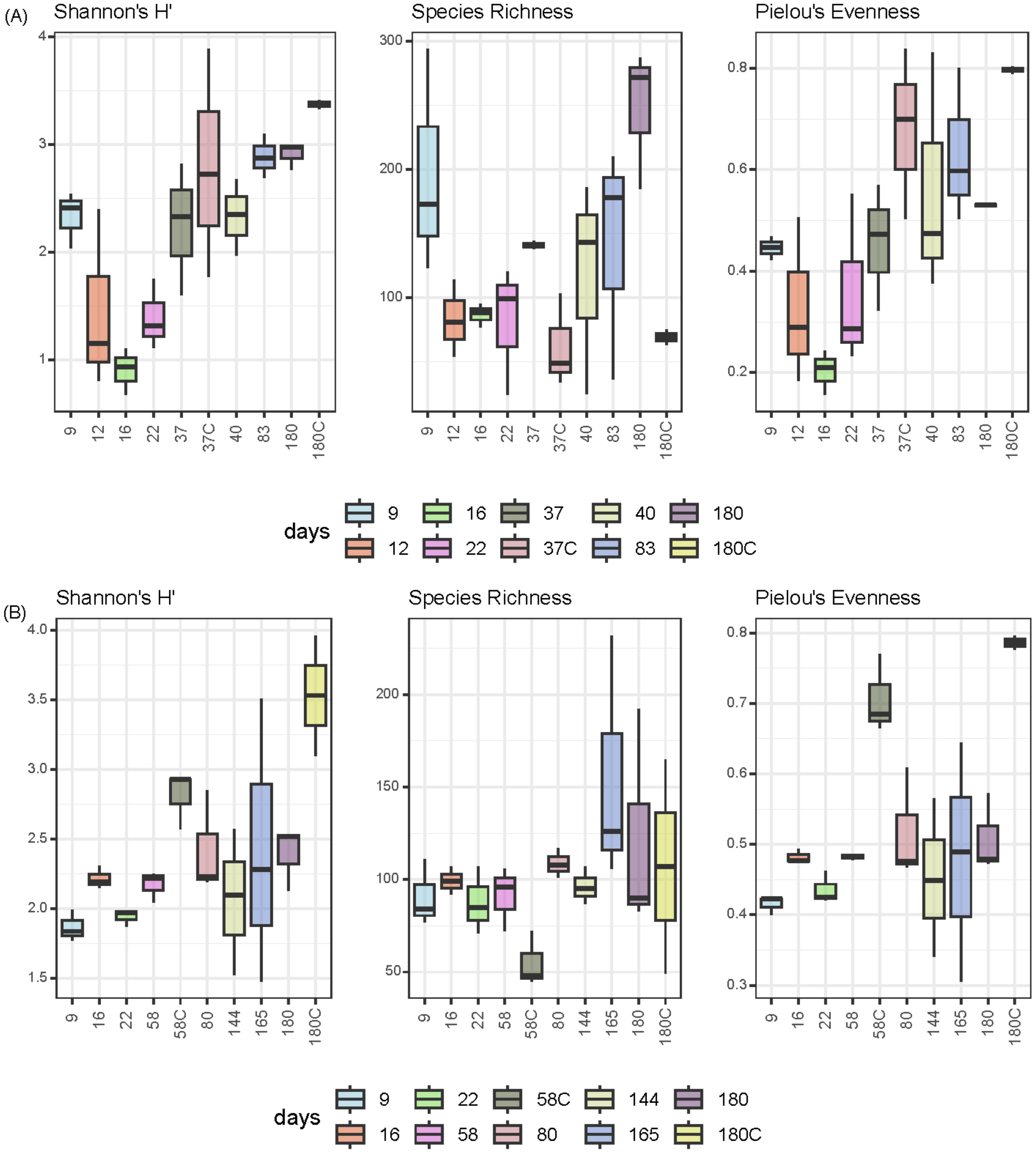

Figure S12. Stacked bar graph of the microbial community composition (top 20 respective taxa with 'Other' depicting the remaining diversity totaling 100%) at the level of domain (A), kingdom (B), phylum (C), class (D), order (E), and family (F) for the parent sediment (Top), USCH samples (middle), and UJA samples (bottom). All samples are biological triplicates. The color scheme should be read left to right, e.g. "Other" to the far left.

(A) Domain

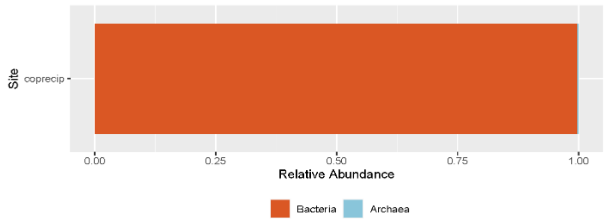

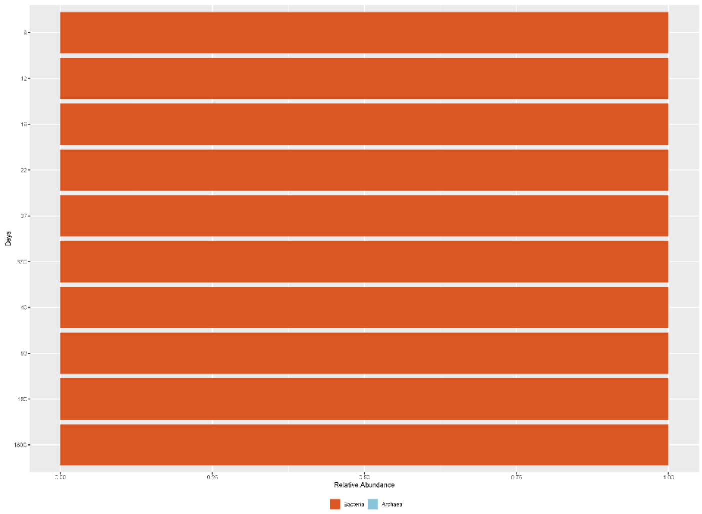

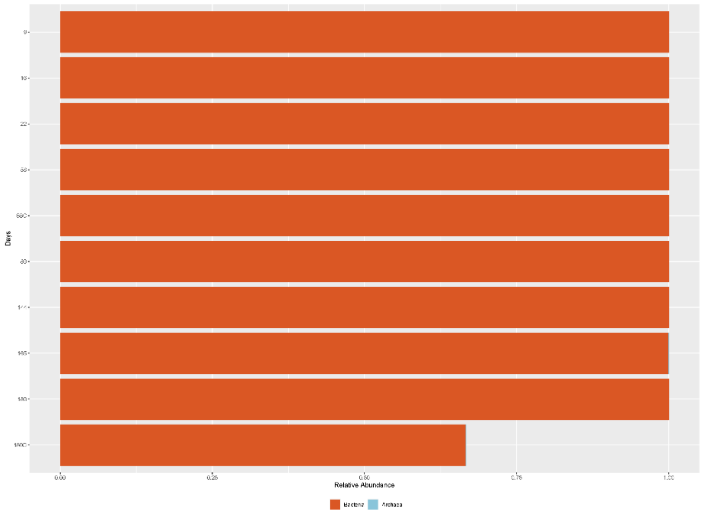

(B) Kingdom

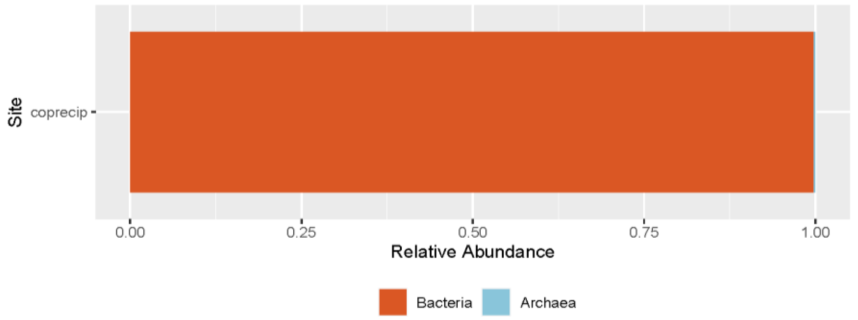
Site
coprecip
0.00
0.25
0.50
0.75
1.00
Relative Abundance
Bacteria
Archaea

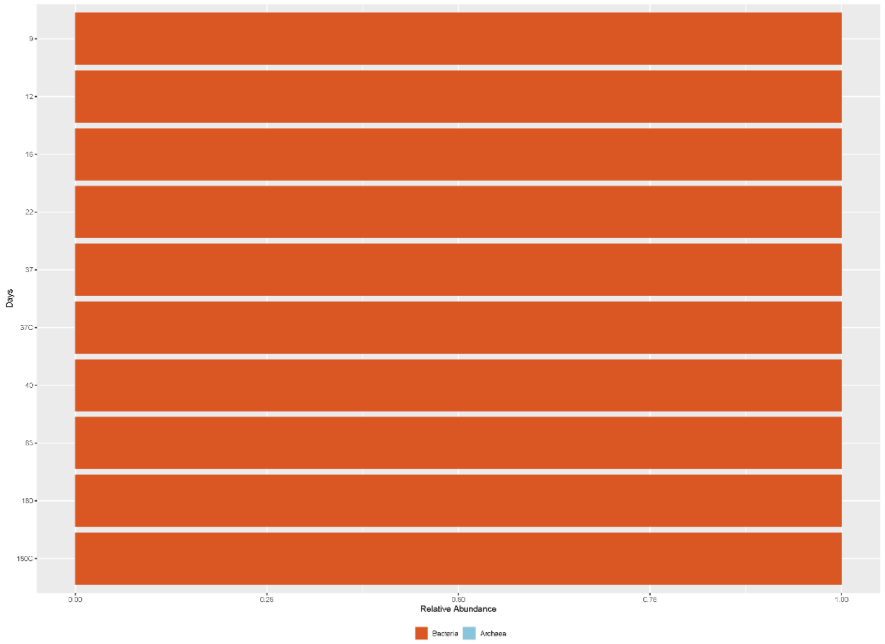
Days
9
12
16
22
37
37C
40
63
180
150C
0.00
Relative Abundance
Bacteria
Archaea

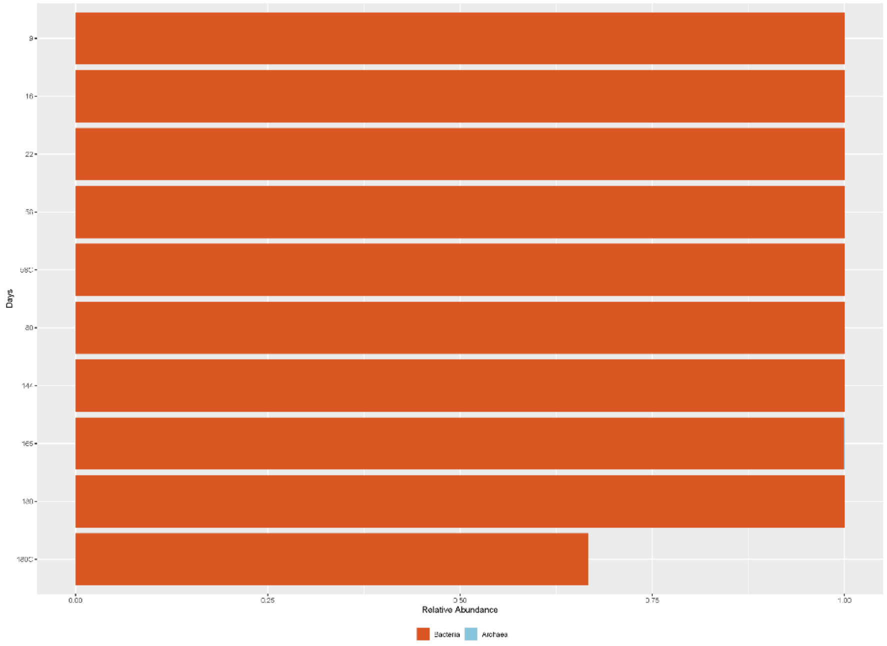
Days
9
16
22
80
144
165
180
180C
0.00
Relative Abundance
Bacteria
Archaea

(C) Phylum

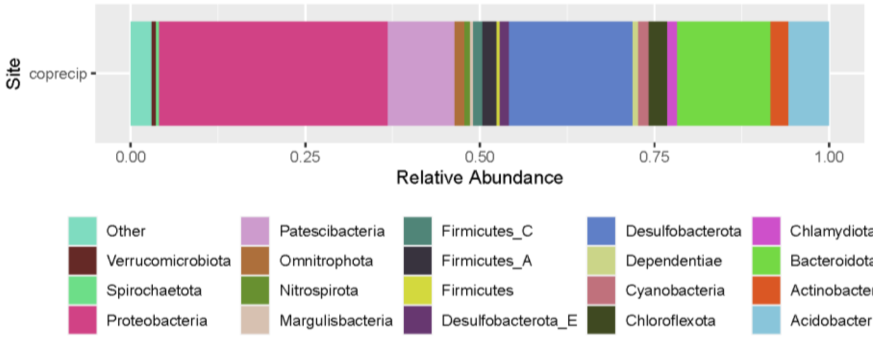
Site
coprecip
0.00
0.25
0.50
0.75
1.00
Relative Abundance
Other
Verrucomicrobiota
Spirochaetota
Proteobacteria
Patescibacteria
Omnitrophota
Nitrospirota
Margulisbacteria
Firmicutes_C
Firmicutes_A
Firmicutes
Desulfobacterota_E
Desulfobacterota
Dependentiae
Cyanobacteria
Chloroflexota

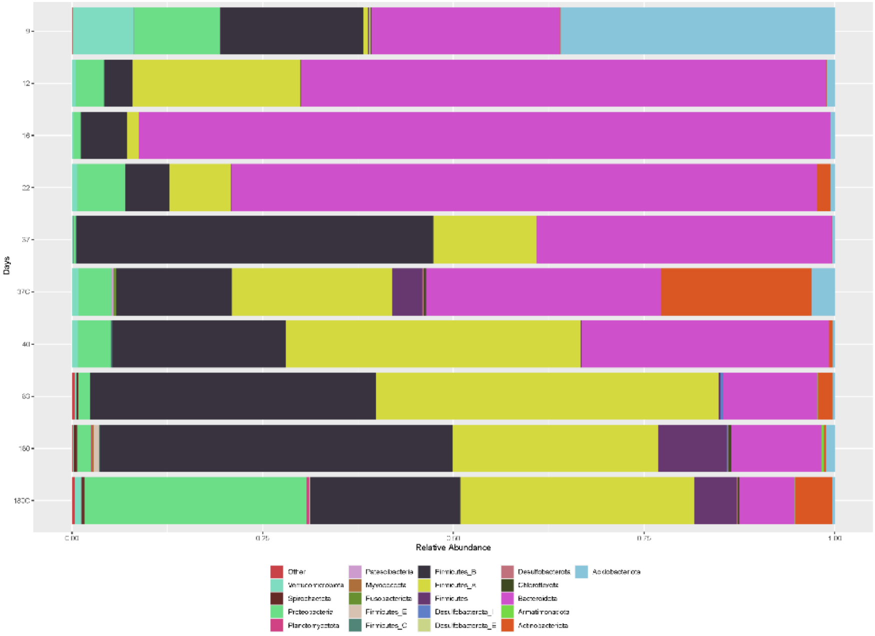
Days
Relative Abundance

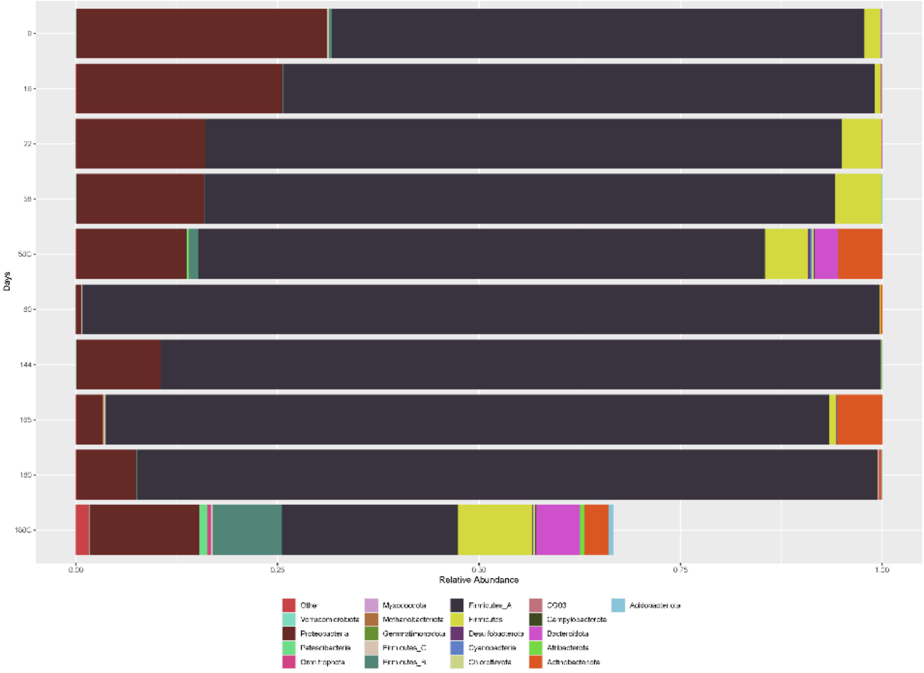
Days
Relative Abundance

(D) Class

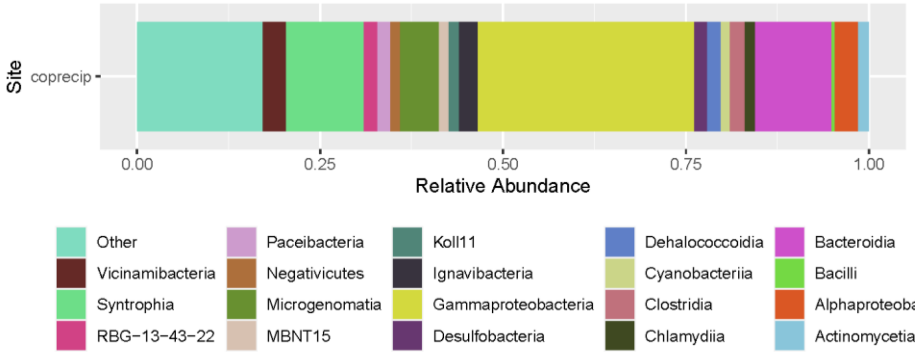
Site
coprecip
0.00
0.25
0.50
0.75
1.00
Relative Abundance
Other
Vicinamibacteria
Syntrophia
RBG−13−43−22
Paceibacteria
Negativicutes
Microgenomatia
MBNT15
Koll11
Ignavibacteria
Gammaproteobacteria
Desulfobacteria
Dehalococcoidia
Cyanobacteriia
Clostridia
Chlamydiia
Bacteroidia
Bacilli
Alphaproteoba
Actinomycetia

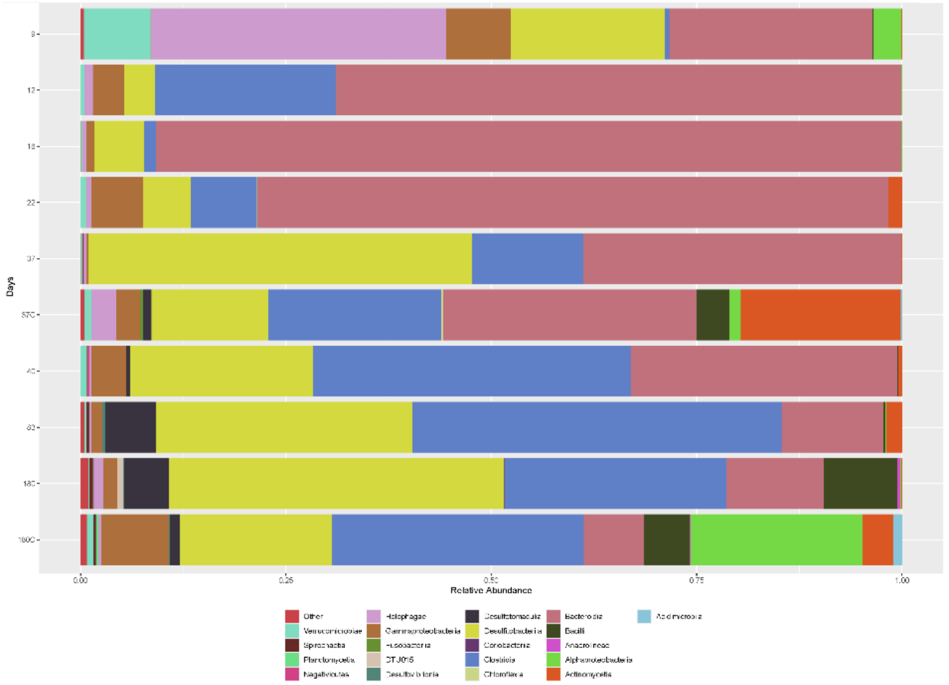

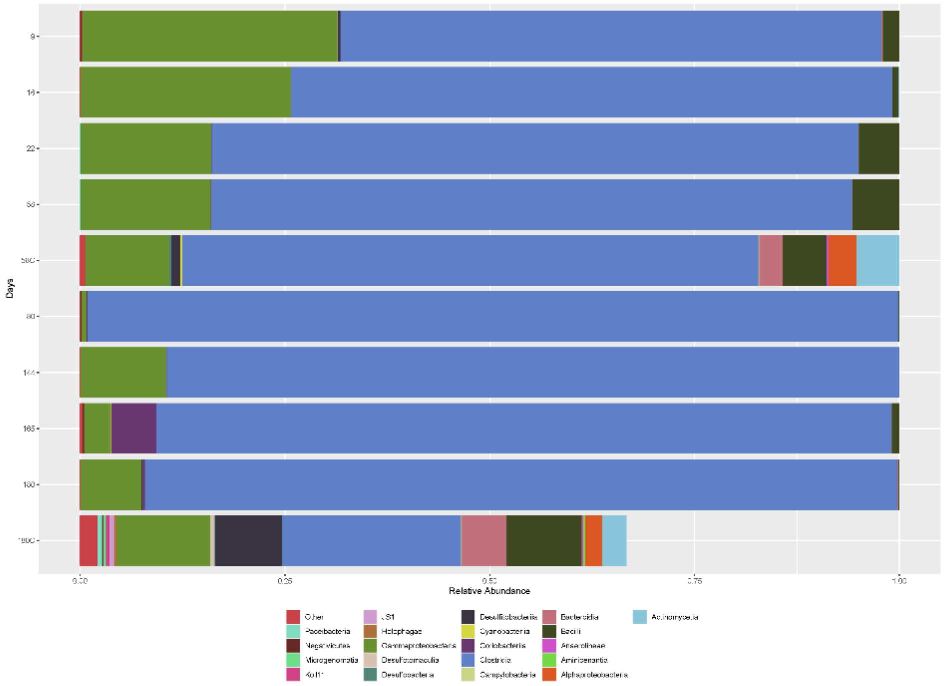

(E) Order

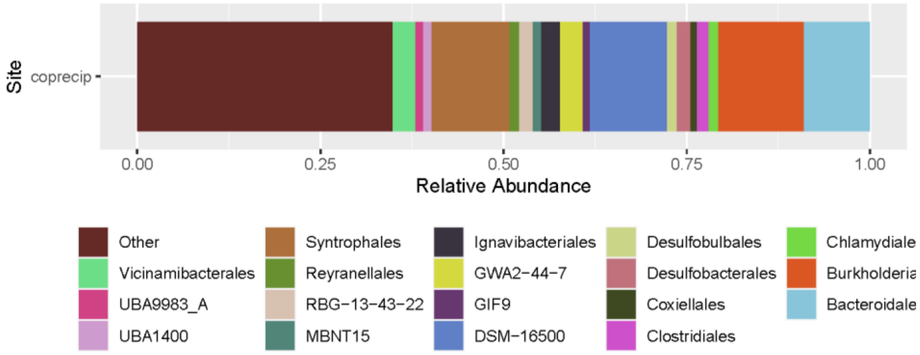
Site
coprecip
0.00
0.25
0.50
0.75
1.00
Relative Abundance
Other
Vicinamibacterales
UBA9983_A
UBA1400
Syntrophales
Reyranellales
RBG−13−43−22
MBNT15
Ignavibacteriales
GWA2−44−7
GIF9
DSM−16500
Desulfobulbales
Desulfobacterales
Coxiellales
Clostridiales
Chlamydiale
Burkholderia
Bacteroidale

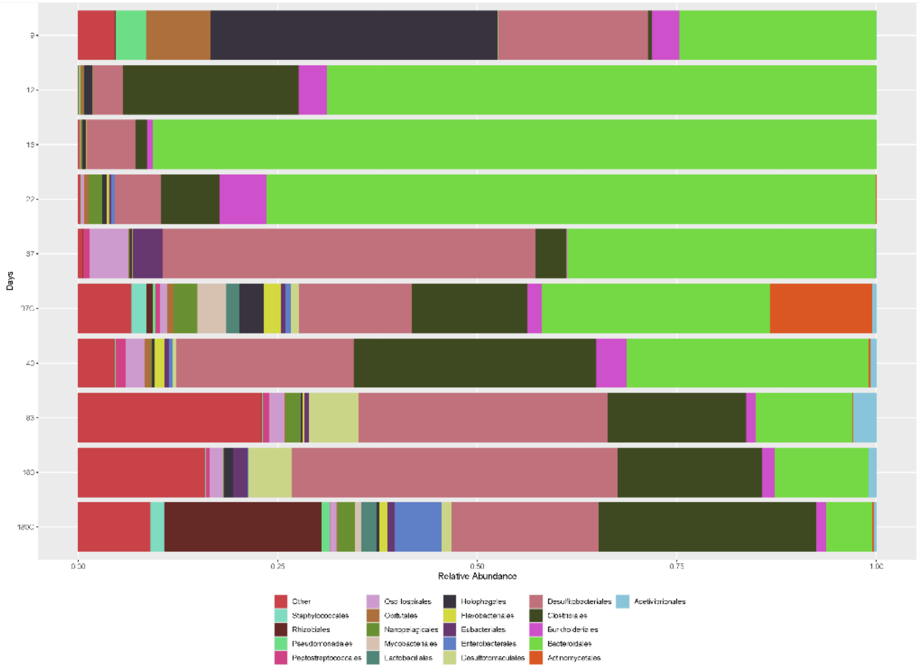
Days
Relative Abundance

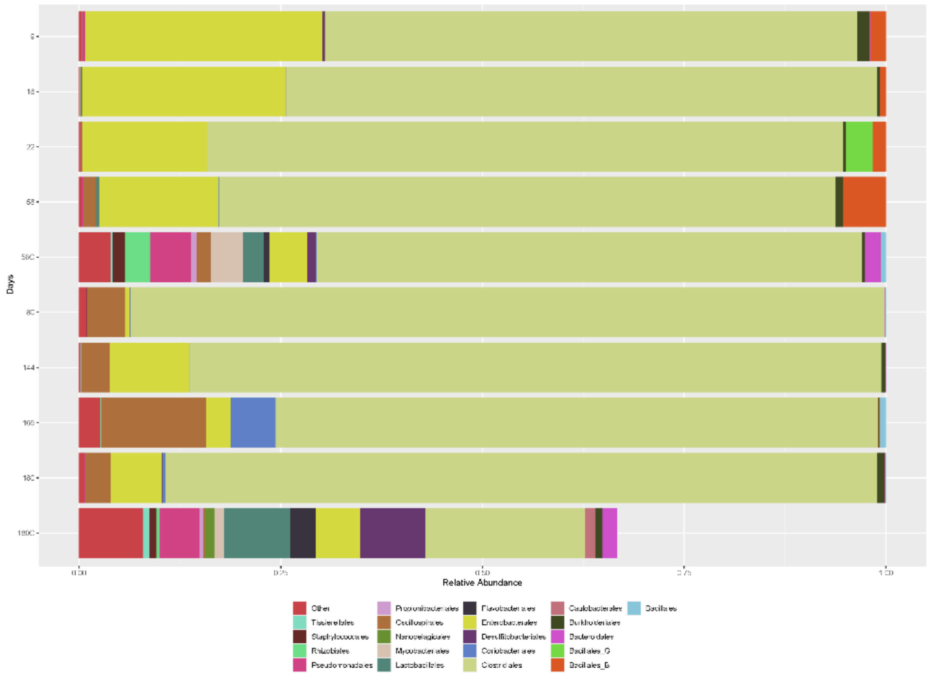
Days
Relative Abundance

(F) Family

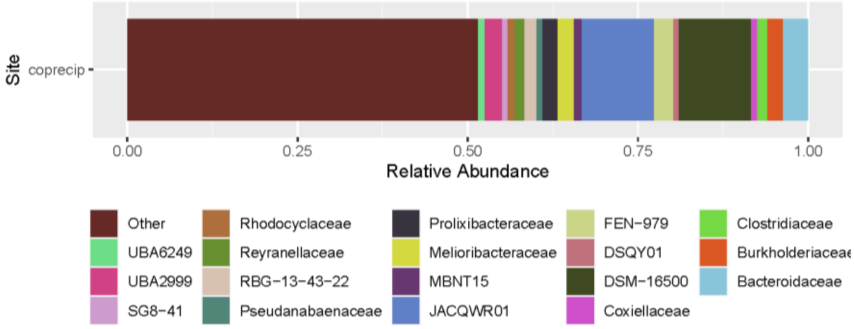

Site
coprecip
0.00
0.25
0.50
0.75
1.00
Relative Abundance
Other
UBA6249
UBA2999
SG8−41
Rhodocyclaceae
Reyranellaceae
RBG−13−43−22
Pseudanabaenaceae
Prolixibacteraceae
Melioribacteraceae
MBNT15
JACQWR01
FEN−979
DSQY01
DSM−16500
Coxiellaceae
Clostridiaceae
Burkholderiaceae
Bacteroidaceae

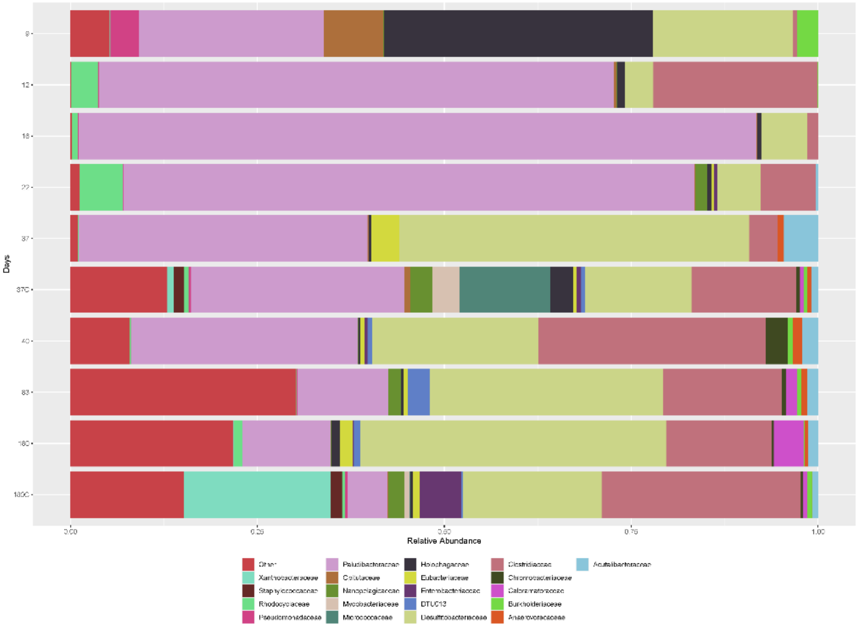

Days
Relative Abundance
Other
Xanthobacteraceae
Rhodocyclaceae
Paludibacteraceae
Mycobacteriaceae
Holophagaceae
Eubacteriaceae
Enterobacteriaceae
DTU013
Clostridiaceae
Chromobacteriaceae
Burkholderiaceae
Acutalibacteraceae

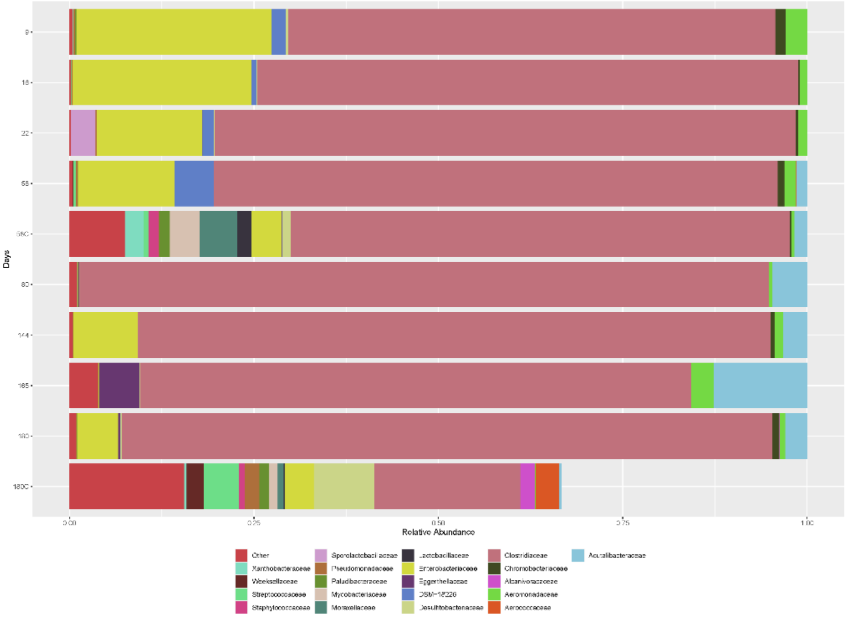

Days
Relative Abundance
Other
Xanthobacteraceae
Weeksellaceae
Streptococcaceae
Staphylococcaceae
Pseudomonadaceae
Paludibacteraceae
Mycobacteriaceae
Moraxellaceae
Lactobacillaceae
Enterobacteriaceae
Eggerthellaceae
DSM−18226
Clostridiaceae
Chromobacteriaceae
Alcanivoracaceae
Aeromonadaceae
Aerococcaceae
Acutalibacteraceae

Table S1. Uranium L_3-edge EXAFS fitting results for the initial materials (UJA and USCH) and selected solid-phase samples of the two incubation experiments.

Sample		U-O_{ax}	U-O_{eq}	U-O_{ax} MS	U-C	U-Fe	U-P	U-O_{eq}-P	ΔE	R-factor[a]
UJA	CN	2.3(3)	6.4(6)	4.6(3)	2.0(7)	1.3(6)			6(2)	0.0027
	R	1.76(1)	2.36(1)	3.52(1)	2.91(3)	3.41(3)				
	σ^2	0.002(1)	0.01*	0.003(8)	0.004*	0.01*				
UJA-9d-2	CN	2.2(5)	5.3(9)	4.4(5)	2.5(11)	0.7(8)			6(5)	0.0062
	R	1.77(2)	2.35(3)	3.54(2)	2.92(4)	3.38(9)				
	σ^2	0.002(2)	0.01*	0.002(2)	0.004*	0.01*				
UJA-22d-2	CN	2.4(6)	4.9(5)	4.8(6)	2.4(12)	0.6(9)			6(4)	0.0069
	R	1.78(2)	2.35(3)	3.55(1)	2.91(4)	3.41(11)				
	σ^2	0.004(2)	0.01*	0.004(2)_	0.004*	0.01*				
UJA-58d-2	CN	2.4(7)	5.1(13)	4.8(7)	2.3(14)	0.7(11)			6(5)	0.0109
	R	1.77(2)	2.34(4)	3.55(2)	2.91(5)	3.43(12)				
	σ^2	0.003(3)	0.01*	0.002(1)	0.004*	0.01*				
UJA-144d-2	CN	2.3(5)	5.3(9)	4.6(5)	2.5(11)	0.7(8)			4(3)	0.0057
	R	1.76(1)	2.32(3)	3.53(1)	2.91(4)	3.53(8)				
	σ^2	0.002(2)	0.01*	0.001(1)	0.004*	0.01*				
UJA-180d-2	CN	2.4(5)	5.0(9)	4.8(5)	2.6(10)	0.9(7)			5(3)	0.0050
	R	1.77(2)	2.34(3)	3.55(2)	2.91(3)	3.41(6)				
	σ^2	0.003(2)	0.01*	0.002(1)	0.004*	0.01*				
USCH	CN	2.5(4)	5.1(7)	5.0(4)	2.6(10)	1.3(8)			8(2)	0.0059
	R	1.77(1)	2.37(2)	3.55(1)	2.93(3)	3.44(4)				
	σ^2	0.003(1)	0.01*	0.001(1)	0.004*	0.01*				
USCH-9d-2	CN	2.5(4)	4.9(7)	5.0(4)	2.7(9)	0.9(7)			7(2)	0.0059
	R	1.78(1)	2.36(2)	3.56(1)	2.93(3)	3.47(6)				
	σ^2	0.003(1)	0.01*	0.002(1)	0.004*	0.01*				
USCH-12d-2	CN	2.3(6)	4.7(10)	4.6(6)	2.6(14)	0.7(10)			6(4)	0.0127
	R	1.77(2)	2.35(4)	3.55(1)	2.92(4)	3.46(11)				
	σ^2	0.003(2)	0.01*	0.002(1)	0.004*	0.01*				
USCH-16d-2	CN	1.9(4)	4.2(7)	3.8(4)	2.4(9)		0.5(6)	1.0(6)	6(3)	0.0156
	R	1.77(2)	2.35(3)	3.54(2)	2.93(3)		3.89(8)	4.12(8)		
	σ^2	0.003(2)	0.01*	0.004(1)_	0.004*		0.003*	0.003*		
USCH-22d-2	CN	1.9(3)	3.7(6)	3.8(3)	1.8(8)		0.5(5)	1.0(5)	8(3)	0.0136
	R	1.78(1)	2.36(3)	3.56(1)	2.91(4)		3.91(6)	4.14(6)		
	σ^2	0.002(1)	0.01*	0.002(1)	0.004*		0.003*	0.003*		
USCH-37d-2	CN	1.9(4)	3.5(7)	3.8(4)	1.9(10)		0.4(6)	0.8(6)	8(4)	0.0218
	R	1.78(2)	2.36(4)	3.56(2)	2.92(4)		3.91(9)	4.14(9)		
	σ^2	0.003(2)	0.01*	0.003(1)	0.004*		0.003*	0.003*		
USCH-83d-2	CN	1.9(3)	3.9(6)	3.8(3)	2.1(9)		0.3(5)	0.6(5)	7(3)	0.0109
	R	1.78(1)	2.36(2)	3.56(1)	2.92(3)		3.91(9)	4.14(9)		
	σ^2	0.003(1)	0.01*	0.003(1)	0.004*		0.003*	0.003*		
USCH-180d-2	CN	1.8(3)	3.5(5)	3.6(3)	2.0(7)		0.5(4)	1.0(4)	8(3)	0.0099
	R	1.78(1)	2.36(3)	3.56(1)	2.92(3)		3.89(6)	4.12(6)		
	σ^2	0.003(1)	0.01*	0.003(1)	0.004*		0.003*	0.003*		

CN, coordination number; R: inter-atomic distance; σ^2, Debye–Waller factor; ΔE, energy shift parameter

The numbers in parentheses are estimated uncertainties for the last digit.

*Fixed parameters

U-O_{ax} MS: sum of three multiple scattering paths, including U-O_{ax1}-O_{ax2} (CN=CN(U-O_{ax}), R=2*R(U-O_{ax}), σ^2=2*σ^2 (U-O_{ax}), U-O_{ax1}-U-O_{ax2} (CN=CN(U-O_{ax}), R=2*R(U-O_{ax}), σ^2=2*σ^2 (U-O_{ax}), U-O_{ax1}-U-O_{ax1} (CN=CN(U-O_{ax}), R=2*R(U-O_{ax}), σ^2=2*σ^2 (U-O_{ax}).

[a]R-factor = $\sum((\text{data-fit})^2/\sum \text{data}^2)$

Table S2. Temporal changes in water chemistry during the incubation experiment with U(VI)-sorbed schwertmannite

	Duration (day)	Sample ID	pH	$Fe(II)_{(aq)}$ mg/L	Fe_{tot} mg/L	$S_{(aq)}$ mg/L	$U_{(aq)}$ mg/L	$P_{(aq)}$ mg/L	$Ca_{(aq)}$ mg/L	$K_{(aq)}$ mg/L	$Mg_{(aq)}$ mg/L	$Na_{(aq)}$ mg/L	$Si_{(aq)}$ mg/L	Percentage (%) of re-liberated U
Samples	9	USCH-9d-triplicate 1	5.1	119	124	697	0.24	2.08	188	414	123	147	4.55	0.47
		USCH-9d-triplicate 2	5.0	137	141	669	0.28	2.13	182	390	119	144	4.61	0.55
		USCH-9d-triplicate 3	5.5	109	112	642	0.27	1.68	198	417	120	152	5.02	0.53
	12	USCH-12d-triplicate 1	5.7	582	608	829	0.18	0.22	197	426	123	158	3.07	0.36
		USCH-12d-triplicate 2	5.6	689	719	936	0.16	0.44	191	422	126	154	2.63	0.31
		USCH-12d-triplicate 3	5.7	785	801	919	0.23	<0.1	185	427	124	151	4.29	0.45
	16	USCH-16d-triplicate 1	4.9	1295	1382	1280	0.64	<0.1	188	414	120	147	2.88	1.27
		USCH-16d-triplicate 2	5.8	870	936	1060	0.25	<0.1	199	427	121	155	2.39	0.50
		USCH-16d-triplicate 3	5.3	1412	1526	1220	0.56	<0.1	186	419	119	145	2.61	1.11
	22	USCH-22d-triplicate 1	5.8	1451	1567	1280	0.40	<0.1	199	409	127	157	2.30	0.78
		USCH-22d-triplicate 2	6.0	1360	1468	1240	0.39	<0.1	195	418	126	155	2.20	0.78
		USCH-22d-triplicate 3	6.0	1391	1503	1220	0.34	<0.1	192	427	125	152	2.26	0.68
	37	USCH-37d-triplicate 1	6.6	822	914	699	0.29	<0.1	177	396	113	147	3.26	0.58
		USCH-37d-triplicate 2	6.8	895	974	736	0.20	<0.1	182	406	116	151	3.51	0.39
		USCH-37d-triplicate 3	6.7	830	873	774	0.29	<0.1	178	426	123	155	3.08	0.57
	40	USCH-40d-triplicate 1	6.7	645	702	618	0.61	<0.1	172	417	119	149	3.36	1.21
		USCH-40d-triplicate 2	6.9	534	589	529	0.59	<0.1	161	407	115	146	2.66	1.17
		USCH-40d-triplicate 3	6.7	578	645	546	0.44	<0.1	158	408	113	146	3.02	0.87
	83	USCH-83d-triplicate 1	7.0	303	329	412	0.21	<0.1	154	419	117	151	2.94	0.41
		USCH-83d-triplicate 2	7.3	212	222	428	0.34	<0.1	131	402	113	148	2.53	0.67
		USCH-83d-triplicate 3	7.1	173	197	419	0.40	<0.1	118	418	113	150	2.67	0.79
	180	USCH-180d-triplicate 1	8.0	4.76	6.25	383	0.26	<0.1	68.2	372	90.6	138	1.08	0.51
		USCH-180d-triplicate 2	8.1	8.53	10.7	317	0.70	<0.1	104	392	101	142	1.30	1.39
		USCH-180d-triplicate 3	8.4	1.98	2.88	262	1.67	<0.1	71.7	379	89.1	140	0.99	3.31
Abiotic controls	37	USCH-C-37d-triplicate 1	4.9	10.3	25.6	584	0.37	2.03	181	405	120	146	6.21	0.73
		USCH-C-37d-triplicate 2	4.7	9.18	35.9	575	0.84	1.49	195	415	121	151	6.18	1.65
		USCH-C-37d-triplicate 3	5.1	8.76	21.4	587	0.22	2.13	197	426	122	154	6.23	0.44
	180	USCH-C-180d-triplicate 1	2.7	40.5	55.7	680	27.4	0.79	211	444	126	160	11.8	54.2
		USCH-C-180d-triplicate 2	2.7	39.3	50.6	719	26.2	0.79	199	436	128	154	12.3	51.9
		USCH-C-180d-triplicate 3	2.8	38.3	47.0	705	25.7	0.85	194	422	125	150	12.1	50.9

Table S3. Temporal changes in water chemistry during the incubation experiment with U(VI)-sorbed jarosite

	Duration (day)	Sample ID	pH	$Fe(II)_{(aq)}$ mg/L	Fe_{tot} mg/L	$S_{(aq)}$ mg/L	$U_{(aq)}$ mg/L	$P_{(aq)}$ mg/L	$Ca_{(aq)}$ mg/L	$K_{(aq)}$ mg/L	$Mg_{(aq)}$ mg/L	$Na_{(aq)}$ mg/L	$Si_{(aq)}$ mg/L	Percentage (%) of re-liberated U
Samples	9	UJA-9d-triplicate 1	3.8	10.7	11.0	472	0.56	7.34	198	156	129	137	25.4	10.1
		UJA-9d-triplicate 2	3.9	10.2	10.3	455	0.12	6.66	210	157	127	142	26.1	2.1
		UJA-9d-triplicate 3	3.8	13.3	16.1	484	0.12	6.55	199	157	130	139	26.0	2.1
	16	UJA-16d-triplicate 1	4.1	70.9	76.0	535	0.11	4.34	205	168	134	139	26.8	2.0
		UJA-16d-triplicate 2	4.0	30.7	33.0	507	0.13	6.16	201	158	130	136	27.0	2.3
		UJA-16d-triplicate 3	4.0	12.1	12.5	492	0.12	6.78	204	160	132	141	27.0	2.3
	22	UJA-22d-triplicate 1	4.0	24.2	27.7	463	0.11	4.89	211	154	128	137	26.3	2.0
		UJA-22d-triplicate 2	3.9	45.5	49.2	494	0.18	5.36	199	162	129	135	27.3	3.2
		UJA-22d-triplicate 3	4.0	30.3	33.8	480	0.10	5.07	198	156	129	134	26.3	1.9
	58	UJA-58d-triplicate 1	4.4	35.7	36.8	500	0.21	3.52	203	157	132	133	27.0	3.9
		UJA-58d-triplicate 2	4.4	42.2	33.3	471	0.09	3.36	215	158	131	137	26.8	1.7
		UJA-58d-triplicate 3	4.2	111	119	525	0.18	4.22	208	176	134	138	28.1	3.2
	80	UJA-80d-triplicate 1	4.7	67.8	73.6	484	0.07	1.76	197	133	131	126	26.6	1.3
		UJA-80d-triplicate 2	4.5	37.7	40.8	451	0.08	2.59	214	129	132	134	25.9	1.5
		UJA-80d-triplicate 3	4.5	40.8	44.3	428	0.09	2.65	213	128	131	133	25.0	1.6
	144	UJA-144d-triplicate 1	3.8	43.6	43.9	493	0.12	3.19	220	160	134	139	27.2	2.2
		UJA-144d-triplicate 2	4.1	57.1	57.7	483	0.09	1.8	215	162	132	136	26.6	1.7
		UJA-144d-triplicate 3	3.8	58.8	60.3	513	0.14	2.77	202	155	131	131	27.5	2.5
	165	UJA-165d-triplicate 1	5.1	52.0	53.7	475	0.10	2.14	200	129	133	128	27.0	1.8
		UJA-165d-triplicate 2	5.0	75.6	78.3	471	0.11	2.67	195	133	130	124	25.8	1.9
		UJA-165d-triplicate 3	4.2	56.1	58.7	521	0.15	2.52	213	164	139	137	26.8	2.7
	180	UJA-180d-triplicate 1	4.4	76.1	78.9	500	0.10	1.32	194	155	127	125	26.5	1.8
		UJA-180d-triplicate 2	4.2	56.7	57.2	502	0.09	1.61	202	155	131	129	26.3	1.7
		UJA-180d-triplicate 3	5.0	163	169	495	0.07	1.47	193	153	129	128	25.0	1.4
Abiotic controls	58	UJA-C-58d-triplicate 1	4.3	1.29	1.45	494	0.10	10.5	194	156	128	131	24.0	1.8
		UJA-C-58d-triplicate 2	4.3	1.20	1.36	463	0.11	10.1	201	155	125	134	23.8	2.0
		UJA-C-58d-triplicate 3	4.3	1.16	1.30	474	0.10	10.4	208	159	128	138	23.4	1.8
	180	UJA-C-180d-triplicate 1	3.8	4.42	4.60	486	0.12	8.82	194	154	129	127	24.4	2.2
		UJA-C-180d-triplicate 2	3.7	4.37	4.56	437	0.13	8.50	196	146	122	125	23.8	2.4
		UJA-C-180d-triplicate 3	3.7	4.35	4.46	500	0.12	9.27	199	158	133	131	24.9	2.3
Blank duplicates of ICP measurements		UJA-9d-triplicate 3				489	0.12	6.47	202	159	132	140	26.1	2.1
		UJA-22d-triplicate 3				486	0.12	6.46	203	160	132	140	26.1	2.3

Table S4. SEM-EDS results of the points marked in Figures S3-9.

Samples	Points	O wt %	C wt %	Fe wt %	S wt %	P wt %	U wt %	Na wt %	Mg wt %	Al wt %	Si wt %	Cl wt %	K wt %	Ca wt %	Molar Fe/S ratio	Molar Fe/K ratio	Sum wt %
UJA-9d-triplicate 2	1	39.0	4.2	24.3	10.6	-	-	-	-	0.1	0.2	-	5.6	-	1.3	3.0	84.1
	2	37.5	3.7	24.9	10.4	-	-	-	-	-	0.2	-	5.5	-	1.4	3.2	82.1
	3	37.8	3.7	24.4	10.7	-	-	-	-	0.1	0.2	-	5.7	-	1.3	3.0	82.6
UJA-180d-triplicate 2	4	43.1	5.8	24.0	10.5	-	-	-	-	0.1	0.2	-	5.7	-	1.3	2.9	89.4
	5	40.0	5.3	24.0	9.4	-	-	-	-	0.2	0.3	-	5.1	-	1.5	3.3	84.4
	6	49.4	8.4	23.3	10.4	-	-	-	-	0.2	0.3	-	5.3	-	1.3	3.1	97.3
	7	52.3	9.5	23.4	10.2	-	-	-	-	0.2	0.2	-	5.3	-	1.3	3.1	101
UJA-180d-C- triplicate 2	8	40.0	5.0	23.5	10.1	-	-	-	-	-	0.3	-	5.3	0.2	1.3	3.1	84.3
	9	44.7	6.4	23.0	10.7	-	-	-	-	-	0.3	-	5.1	-	1.2	3.1	90.7
	10	36.9	3.8	22.4	10.6	-	-	-	-	-	0.3	-	5.4	-	1.2	2.9	79.3
	11	45.4	7.0	22.6	10.4	-	-	-	-	-	0.3	-	5.1	-	1.2	3.1	90.9
USCH-9d-triplicate 2	12	32.6	4.1	34.0	3.0	-	0.2	-	-	-	2.0	-	0.3	0.6	6.5	79	76.9
	13	35.0	4.3	25.3	2.4	0.1	0.3	0.9	0.3	0.3	6.4	-	0.3	1.7	6.0	59	77.2
	14	36.3	5.3	33.5	3.2	0.1	0.2	-	-	-	2.2	0.1	0.3	0.6	6.0	78	81.8
	15	38.4	5.8	34.8	3.4	-	0.2	-	-	-	2.4	-	0.3	0.6	5.8	81	85.9
USCH-12d-triplicate 2	16	43.9	8.1	33.5	3.0	0.1	0.4	1.2	0.7	0.2	1.8	-	0.4	0.6	6.4	58	93.8
	17	49.2	9.3	31.0	3.1	0.1	0.3	1.5	0.8	0.2	4.0	-	0.4	1.2	5.7	54	101
	18	51.8	9.2	29.2	3.2	-	0.4	2.4	0.9	0.3	6.8	-	0.4	1.4	5.2	51	106
USCH-180d-triplicate 2	19	22.3	2.0	35.5	0.7	-	0.2	-	-	-	0.4	-	0.1	0.3	29	247	61.5
	20	31.7	4.9	37.8	1.2	0.1	0.3	-	-	-	0.4	-	0.3	0.4	18	88	77.0
	21	38.5	6.8	36.7	2.5	0.1	0.3	-	-	-	0.4	-	0.3	0.4	8.4	85	86.0
	22	25.8	3.0	36.3	1.0	0.1	0.3	-	-	-	0.4	-	0.2	0.4	21	126	67.5
	23	43.7	6.5	35.5	6.8	0.1	0.2	-	-	0.2	0.3	-	0.3	0.4	3.0	82	93.9
	24	36.6	5.8	34.2	3.7	0.1	-	-	-	0.2	0.4	-	0.2	0.3	5.3	119	81.6
	25	21.5	2.4	29.0	0.7	-	0.1	-	-	-	0.4	-	0.1	2.6	24	202	56.8
USCH-180d-C- triplicate 2	26	40.7	6.2	32.8	3.6	-	-	-	-	-	3.7	0.2	0.2	0.8	5.2	114	88.3
	27	41.2	6.9	32.9	3.3	0.2	0.1	-	-	-	2.8	0.1	0.2	0.7	5.7	115	88.4
	28	44.6	6.9	34.5	4.3	-	-	-	-	-	4.0	0.2	0.3	0.9	4.6	80	95.6

-not detected

Table S5. Details of sampling, Illumina sequencing, and data processing.

Sample	Unique sample ID	Sequencing identifier	DNA extraction concentration [ng/µL]	Kreads	>=Q30 (%)	DADA2 Input sequences	DADA2 Filtered sequences	ASVs
USCH-9d-triplicate_1	USCH.1.1	P28659_1001	3.56	333.87	77.28	330691	203688	164333
USCH-9d-triplicate_2	USCH.1.2	P28659_1002	1.26	338.67	76.87	335016	206857	132080
USCH-9d-triplicate_3	USCH.1.3	P28659_1003	1.23	430.27	77.51	424968	263282	233801
USCH-12d-triplicate_1	USCH.2.1	P28659_1004	>60	275.3	78.23	272672	173459	144104
USCH-12d-triplicate_2	USCH.2.2	P28659_1005	60	313.48	79.29	310665	203322	186889
USCH-12d-triplicate_3	USCH.2.3	P28659_1006	4.13	328.18	80.54	325025	217895	154520
USCH-16d-triplicate_1	USCH.3.1	P28659_1007	>60	353.88	79.21	350610	227793	206789
USCH-16d-triplicate_2	USCH.3.2	P28659_1008	>60	394.55	79.32	390607	255421	236129
USCH-16d-triplicate_3	USCH.3.3	P28659_1009	>60	327.84	79.03	324960	211224	183550
USCH-22d-triplicate_1	USCH.4.1	P28659_1010	>60	530.02	79.13	523921	340337	296542
USCH-22d-triplicate_2	USCH.4.2	P28659_1011	>60	490.58	78.89	485524	313404	248089
USCH-22d-triplicate_3	USCH.4.3	P28659_1012	17.4	5.18	72.38	5050	2548	1777
USCH-37d-triplicate_1	USCH.5.1	P28659_1013	21	420.77	76.85	417232	254147	189606
USCH-37d-triplicate_2	USCH.5.2	P28659_1014	17.4	516.97	76.99	512539	314601	243002
USCH-37d-triplicate_3	USCH.5.3	P28659_1015	25.8	306.23	78.56	303524	193897	161138
USCH-C-37d-triplicate_1	USCH.5.1.C	P28659_1016	0.146	9.56	72.66	9370	4619	3516
USCH-C-37d-triplicate-2	USCH.5.2.C	P28659_1017	0.078	9.81	72.3	9608	4867	3201
USCH-C-37d-triplicate_3	USCH.5.3.C	P28659_1018	0.072	13.11	73.74	12821	6806	5387
USCH-40d-triplicate_1	USCH.6.1	P28659_1019	60	284.9	78.75	281770	180270	123683
USCH-40d-triplicate_2	USCH.6.2	P28659_1020	12	2.88	71.82	2822	1360	758
USCH-40d-triplicate_3	USCH.6.3	P28659_1021	9.78	340.76	79.29	338174	218917	165541
USCH-83d-triplicate_1	USCH.7.1	P28659_1022	13	423.44	76.84	420049	254635	178057
USCH-83d-triplicate_2	USCH.7.2	P28659_1023	20.4	331.1	77.4	328325	201089	106941
USCH-83d-triplicate_3	USCH.7.3	P28659_1024	6.82	5.18	72.21	5074	2482	1503
USCH-180d-triplicate_1	USCH.8.1	P28659_1025	4.66	285.29	75.68	282228	165460	102446
USCH-180d-triplicate_2	USCH.8.2	P28659_1026	5.93	657.39	76.16	650299	388255	266489
USCH-180d-triplicate_3	USCH.8.3	P28659_1027	13.9	898.13	77.62	887602	554216	422930
USCH-C-180d-triplicate_1	USCH.8.1.C	P28659_1028	0.061	8.79	72.93	8596	4460	3453
USCH-C-180d-triplicate_2	USCH.8.2.C	P28659_1029	<0.05	11.03	72.12	10796	5313	3625
USCH-C-180d-triplicate_3	USCH.8.3.C	P28659_1030	0.164	11.24	71.36	11018	5191	3410

UJA-9d-triplicate_1	UJA-LPH.1.1	P28659_1031	27.9	326.26	76.63	322905	195814	162984
UJA-9d-triplicate_2	UJA-LPH.1.2	P28659_1032	>60	268.24	78.84	265015	163325	84360
UJA-9d-triplicate_3	UJA-LPH.1.3	P28659_1033	21	687.09	80.46	680459	456300	352719
UJA-16d-triplicate_1	UJA-LPH.2.1	P28659_1034	9.55	382.75	80.56	378485	246575	131641
UJA-16d-triplicate_2	UJA-LPH.2.2	P28659_1035	17.5	464.36	79.35	459565	293339	209511
UJA-16d-triplicate_3	UJA-LPH.2.3	P28659_1036	19.2	575.86	78.92	569717	354813	245886
UJA-22d-triplicate_1	UJA-LPH.3.1	P28659_1037	8.12	593.75	80.7	588118	398311	327285
UJA-22d-triplicate_2	UJA-LPH.3.2	P28659_1038	6.01	517.09	78.66	510872	313156	229951
UJA-22d-triplicate_3	UJA-LPH.3.3	P28659_1039	2.48	357.48	79.66	353929	231930	182780
UJA-58d-triplicate_1	UJA-LPH.4.1	P28659_1040	9.11	567.92	80.11	561551	373734	263671
UJA-58d-triplicate_2	UJA-LPH.4.2	P28659_1041	11.9	406.27	80.18	401310	267502	198434
UJA-58d-triplicate_3	UJA-LPH.4.3	P28659_1042	10	399.07	78.13	391547	217508	161172
UJA-C-58d-triplicate_1	UJA-LPH.4.1.C	P28659_1043	0.08	12.44	73.69	11463	5752	3952
UJA-C-58d-triplicate_2	UJA-LPH.4.2.C	P28659_1044	0.059	6.42	73.82	5684	3131	2558
UJA-C-58d-triplicate_3	UJA-LPH.4.3.C	P28659_1045	<0.05	10	74.1	9379	4846	3534
UJA-80d-triplicate_1	UJA-LPH.5.1	P28659_1046	55	342.26	80.55	338436	224206	138536
UJA-80d-triplicate_2	UJA-LPH.5.2	P28659_1047	13.2	443.24	80.78	438549	295736	244955
UJA-80d-triplicate_3	UJA-LPH.5.3	P28659_1048	46.3	445.83	81.24	441042	300872	217835
UJA-144d-triplicate_1	UJA-LPH.6.1	P28659_1049	11.7	450.07	80.63	445023	301459	269075
UJA-144d-triplicate_2	UJA-LPH.6.2	P28659_1050	10.7	355.68	78.73	350248	203705	125235
UJA-144d-triplicate_3	UJA-LPH.6.3	P28659_1051	11.2	563.11	80.88	556389	375862	309998
UJA-165d-triplicate_1	UJA-LPH.7.1	P28659_1052	>60	447.25	79.64	442134	291526	180504
UJA-165d-triplicate_2	UJA-LPH.7.2	P28659_1053	0.819	435.37	77.26	430447	263433	235177
UJA-165d-triplicate_3	UJA-LPH.7.3	P28659_1054	9.41	610.9	80.84	603926	411224	373589
UJA-180d-triplicate_1	UJA-LPH.8.1	P28659_1055	18.4	394.35	78.86	388536	230496	151134
UJA-180d-triplicate_2	UJA-LPH.8.2	P28659_1056	12.7	462.77	80.12	457036	302071	195839
UJA-180d-triplicate_3	UJA-LPH.8.3	P28659_1057	19.9	445.7	80.64	441063	298447	233932
UJA-C-180d-triplicate_1	UJA-LPH.8.1.C	P28659_1058	0.2	32.85	74.43	32239	17087	13795
UJA-C-180d-triplicate_3	UJA-LPH.8.3.C	P28659_1060	<0.05	5.51	70.72	5316	2470	1482
Parent-sediment_1	Parent-sediment_1	P30102_1067	0.27	0.02	64.9	7	2	1
Parent-sediment_2	Parent-sediment_2	P30102_1068	0.32	0.01	70.82	5779	3331	2854
Parent-sediemnt_3	Parent-sediemnt_3	P30102_1069	0.17	5.87	71.74	6817	4123	3484

Table S6. One-way ANOVA results for three microbial diversity indices (Shannon's diversity, species richness, and Pielou's evenness) of the microbial community in the SCH samples.

		Degree of freedom	Sum of Square	Mean Square	F value	Probability (>F)
Shannon's diversity	Days	9	16.71	1.856	7.141	0.00013***
	Residuals	20	5.20	0.260		
Species richness	Days	9	99171	11019	3.534	0.0090**
	Residuals	20	62368	3118		
Pielou's evenness	Days	9	0.8599	0.0955	5.221	0.0010**
	Residuals	20	0.3660	0.0183		

***significance at the 0.001 level; ** significance at the 0.01 level; *significance at the 0.05 level

Table S7. Results of Tukey's Post-hoc tests for mean differences in Shannon's diversity between the USCH samples.

	Mean difference	95% confidence interval		Adjusted *p* value
		Lower bound	Upper bound	
USCH-12d vs USCH-9d	-0.8748	-2.3490	0.5994	0.5464
USCH-16d vs USCH-9d	-1.4205	-2.8947	0.0538	0.0648
USCH-22d vs USCH-9d	-0.9332	-2.4074	0.5410	0.4628
USCH-37d vs USCH-9d	-0.0772	-1.5515	1.3970	1.0000
USCH-C-37d vs USCH-9d	0.4643	-1.0100	1.9385	0.9771
USCH-40d vs USCH-9d	0.0033	-1.4710	1.4775	1.0000
USCH-83d vs USCH-9d	0.5580	-0.9163	2.0322	0.9316
USCH-180d vs USCH-9d	0.5817	-0.8925	2.0560	0.9143
USCH-C-180d vs USCH-9d	1.0427	-0.4315	2.5169	0.3227
USCH-16d vs USCH-12d	-0.5457	-2.0199	0.9286	0.9396
USCH-22d vs USCH-12d	-0.0584	-1.5326	1.4158	1.0000
USCH-37d vs USCH-12d	0.7976	-0.6767	2.2718	0.6597
USCH-C-37d vs USCH-12d	1.3391	-0.1352	2.8133	0.0949
USCH-40d vs USCH-12d	0.8780	-0.5962	2.3523	0.5417
USCH-83d vs USCH-12d	1.4328	-0.0415	2.9070	0.0611
USCH-180d vs USCH-12d	1.4565	-0.0177	2.9308	0.0545
USCH-C-180d vs USCH-12d	1.9175	0.4433	3.3917	0.0052
USCH-22d vs USCH-16d	0.4873	-0.9870	1.9615	0.9690
USCH-37d vs USCH-16d	1.3432	-0.1310	2.8175	0.0931
USCH-C-37d vs USCH-16d	1.8847	0.4105	3.3590	0.0062
USCH-40d vs USCH-16d	1.4237	-0.0505	2.8979	0.0638

USCH-83d vs USCH-16d	1.9784	0.5042	3.4527	0.0038
USCH-180d vs USCH-16d	2.0022	0.5280	3.4764	0.0033
USCH-C-180d vs USCH-16d	2.4632	0.9889	3.9374	0.0003
USCH-37d vs USCH-22d	0.8560	-0.6182	2.3302	0.5740
USCH-C-37d vs USCH-22d	1.3975	-0.0768	2.8717	0.0723
USCH-40d vs USCH-22d	0.9365	-0.5378	2.4107	0.4583
USCH-83d vs USCH-22d	1.4912	0.0170	2.9654	0.0460
USCH-180d vs USCH-22d	1.5149	0.0407	2.9892	0.0410
USCH-C-180d vs USCH-22d	1.9759	0.5017	3.4501	0.0038
USCH-C-37d vs USCH-37d	0.5415	-0.9328	2.0157	0.9421
USCH-40d vs USCH-37d	0.0805	-1.3938	1.5547	1.0000
USCH-83d vs USCH-37d	0.6352	-0.8390	2.1094	0.8665
USCH-180d vs USCH-37d	0.6589	-0.8153	2.1332	0.8414
USCH-C-180d vs USCH-37d	1.1199	-0.3543	2.5941	0.2419
USCH-40d vs USCH-C-37d	-0.4610	-1.9353	1.0132	0.9781
USCH-83d vs USCH-C-37d	0.0937	-1.3805	1.5680	1.0000
USCH-180d vs USCH-C-37d	0.1175	-1.3568	1.5917	1.0000
USCH-C-180d vs USCH-C-37d	0.5784	-0.8958	2.0527	0.9169
USCH-83d vs USCH-40d	0.5547	-0.9195	2.0290	0.9337
USCH-180d vs USCH-40d	0.5785	-0.8958	2.0527	0.9168
USCH-C-180d vs USCH-40d	1.0394	-0.4348	2.5137	0.3264
USCH-180d vs USCH-83d	0.0237	-1.4505	1.4980	1.0000
USCH-C-180d vs USCH-83d	0.4847	-0.9895	1.9589	0.9700
USCH-C-180d vs USCH-180d	0.4610	-1.0133	1.9352	0.9781

Table S8. Results of Tukey's Post-hoc tests for mean differences in species richness between the USCH samples.

	Mean difference	95% confidence interval		Adjusted *p* value
		Lower bound	Upper bound	
USCH-12d vs USCH-9d	-113.7	-275.1	47.8	0.3283
USCH-16d vs USCH-9d	-110.0	-271.5	51.5	0.3686
USCH-22d vs USCH-9d	-115.7	-277.1	45.8	0.3075
USCH-37d vs USCH-9d	-55.7	-217.1	105.8	0.9599
USCH-C-37d vs USCH-9d	-134.7	-296.1	26.8	0.1543
USCH-40d vs USCH-9d	-78.7	-240.1	82.8	0.7698
USCH-83d vs USCH-9d	-55.3	-216.8	106.1	0.9614
USCH-180d vs USCH-9d	51.3	-110.1	212.8	0.9757

USCH-C-180d vs USCH-9d	-127.7	-289.1	33.8	0.2016
USCH-16d vs USCH-12d	3.7	-157.8	165.1	1.0000
USCH-22d vs USCH-12d	-2.0	-163.5	159.5	1.0000
USCH-37d vs USCH-12d	58.0	-103.5	219.5	0.9490
USCH-C-37d vs USCH-12d	-21.0	-182.5	140.5	1.0000
USCH-40d vs USCH-12d	35.0	-126.5	196.5	0.9984
USCH-83d vs USCH-12d	58.3	-103.1	219.8	0.9473
USCH-180d vs USCH-12d	165.0	3.5	326.5	0.0427
USCH-C-180d vs USCH-12d	-14.0	-175.5	147.5	1.0000
USCH-22d vs USCH-16d	-5.7	-167.1	155.8	1.0000
USCH-37d vs USCH-16d	54.3	-107.1	215.8	0.9654
USCH-C-37d vs USCH-16d	-24.7	-186.1	136.8	0.9999
USCH-40d vs USCH-16d	31.3	-130.1	192.8	0.9993
USCH-83d vs USCH-16d	54.7	-106.8	216.1	0.9641
USCH-180d vs USCH-16d	161.3	-0.1	322.8	0.0503
USCH-C-180d vs USCH-16d	-17.7	-179.1	143.8	1.0000
USCH-37d vs USCH-22d	60.0	-101.5	221.5	0.9382
USCH-C-37d vs USCH-22d	-19.0	-180.5	142.5	1.0000
USCH-40d vs USCH-22d	37.0	-124.5	198.5	0.9975
USCH-83d vs USCH-22d	60.3	-101.1	221.8	0.9362
USCH-180d vs USCH-22d	167.0	5.5	328.5	0.0390
USCH-C-180d vs USCH-22d	-12.0	-173.5	149.5	1.0000
USCH-C-37d vs USCH-37d	-79.0	-240.5	82.5	0.7658
USCH-40d vs USCH-37d	-23.0	-184.5	138.5	0.9999
USCH-83d vs USCH-37d	0.3	-161.1	161.8	1.0000
USCH-180d vs USCH-37d	107.0	-54.5	268.5	0.4036
USCH-C-180d vs USCH-37d	-72.0	-233.5	89.5	0.8431
USCH-40d vs USCH-C-37d	56.0	-105.5	217.5	0.9585
USCH-83d vs USCH-C-37d	79.3	-82.1	240.8	0.7618
USCH-180d vs USCH-C-37d	186.0	24.5	347.5	0.0162
USCH-C-180d vs USCH-C-37d	7.0	-154.5	168.5	1.0000
USCH-83d vs USCH-40d	23.3	-138.1	184.8	0.9999
USCH-180d vs USCH-40d	130.0	-31.5	291.5	0.1847
USCH-C-180d vs USCH-40d	-49.0	-210.5	112.5	0.9820
USCH-180d vs USCH-83d	106.7	-54.8	268.1	0.4076
USCH-C-180d vs USCH-83d	-72.3	-233.8	89.1	0.8397
USCH-C-180d vs USCH-180d	-179.0	-340.5	-17.5	0.0225

Table S9. Results of Tukey's Post-hoc tests for mean differences in Pielou's evenness between the USCH samples.

	Mean difference	95% confidence interval		Adjusted *p* value
		Lower bound	Upper bound	
USCH-12d vs USCH-9d	-0.1193	-0.5104	0.2719	0.9814
USCH-16d vs USCH-9d	-0.2429	-0.6340	0.1483	0.4878
USCH-22d vs USCH-9d	-0.0888	-0.4799	0.3024	0.9977
USCH-37d vs USCH-9d	0.0093	-0.3818	0.4004	1.0000
USCH-C-37d vs USCH-9d	0.2341	-0.1570	0.6253	0.5352
USCH-40d vs USCH-9d	0.1147	-0.2765	0.5058	0.9856
USCH-83d vs USCH-9d	0.1882	-0.2030	0.5793	0.7814
USCH-180d vs USCH-9d	0.0838	-0.3074	0.4749	0.9985
USCH-C-180d vs USCH-9d	0.3509	-0.0402	0.7421	0.1023
USCH-16d vs USCH-12d	-0.1236	-0.5147	0.2675	0.9766
USCH-22d vs USCH-12d	0.0305	-0.3606	0.4217	1.0000
USCH-37d vs USCH-12d	0.1286	-0.2626	0.5197	0.9700
USCH-C-37d vs USCH-12d	0.3534	-0.0377	0.7446	0.0979
USCH-40d vs USCH-12d	0.2340	-0.1572	0.6251	0.5362
USCH-83d vs USCH-12d	0.3075	-0.0837	0.6986	0.2072
USCH-180d vs USCH-12d	0.2031	-0.1881	0.5942	0.7060
USCH-C-180d vs USCH-12d	0.4702	0.0790	0.8613	0.0111
USCH-22d vs USCH-16d	0.1541	-0.2370	0.5453	0.9150
USCH-37d vs USCH-16d	0.2522	-0.1390	0.6433	0.4390
USCH-C-37d vs USCH-16d	0.4770	0.0859	0.8682	0.0097
USCH-40d vs USCH-16d	0.3576	-0.0336	0.7487	0.0912
USCH-83d vs USCH-16d	0.4311	0.0399	0.8222	0.0236
USCH-180d vs USCH-16d	0.3267	-0.0645	0.7178	0.1533
USCH-C-180d vs USCH-16d	0.5938	0.2026	0.9849	0.0010
USCH-37d vs USCH-22d	0.0981	-0.2931	0.4892	0.9952
USCH-C-37d vs USCH-22d	0.3229	-0.0682	0.7141	0.1628
USCH-40d vs USCH-22d	0.2034	-0.1877	0.5946	0.7039
USCH-83d vs USCH-22d	0.2769	-0.1142	0.6681	0.3214
USCH-180d vs USCH-22d	0.1725	-0.2186	0.5637	0.8508
USCH-C-180d vs USCH-22d	0.4397	0.0485	0.8308	0.0200
USCH-C-37d vs USCH-37d	0.2249	-0.1663	0.6160	0.5866
USCH-40d vs USCH-37d	0.1054	-0.2858	0.4965	0.9919
USCH-83d vs USCH-37d	0.1789	-0.2123	0.5700	0.8241
USCH-180d vs USCH-37d	0.0745	-0.3167	0.4656	0.9994
USCH-C-180d vs USCH-37d	0.3416	-0.0495	0.7328	0.1198
USCH-40d vs USCH-C-37d	-0.1195	-0.5106	0.2717	0.9812

USCH-83d vs USCH-C-37d	-0.0460	-0.4371	0.3452	1.0000
USCH-180d vs USCH-C-37d	-0.1504	-0.5415	0.2408	0.9255
USCH-C-180d vs USCH-C-37d	0.1168	-0.2744	0.5079	0.9838
USCH-83d vs USCH-40d	0.0735	-0.3176	0.4646	0.9995
USCH-180d vs USCH-40d	-0.0309	-0.4221	0.3602	1.0000
USCH-C-180d vs USCH-40d	0.2362	-0.1549	0.6274	0.5238
USCH-180d vs USCH-83d	-0.1044	-0.4956	0.2867	0.9924
USCH-C-180d vs USCH-83d	0.1627	-0.2284	0.5539	0.8876
USCH-C-180d vs USCH-180d	0.2671	-0.1240	0.6583	0.3655

Table S10. One-way ANOVA results for three microbial diversity indices (Shannon's diversity, species richness, and Pielou's evenness) of the microbial community in the UJA samples.

		Degree of freedom	Sum of Square	Mean Square	F value	Probability (>F)
Shannon's diversity	Days	9	5.055	0.5617	3.012	0.0206*
	Residuals	19	3.543	0.1865		
Species richness	Days	9	17726	1970	1.432	0.2430
	Residuals	19	26139	1376		
Pielou's evenness	Days	9	0.3277	0.0364	6.340	0.0004***
	Residuals	19	0.1091	0.0057		

***significance at the 0.001 level; *significance at the 0.05 level

Table S11. Results of Tukey's Post-hoc tests for mean differences in Shannon's diversity between the UJA samples.

	Mean Difference	95% confidence interval		adjusted *p* vale
		lower bound	upper bound	
UJA-16d vs UJA-9d	0.3496	-0.9064	1.6056	0.9893
UJA-22d vs UJA-9d	0.0754	-1.1806	1.3313	1.0000
UJA-58d vs UJA-9d	0.3050	-0.9510	1.5610	0.9959
UJA-C-58d vs UJA-9d	0.9433	-0.3127	2.1993	0.2505
UJA-80d vs UJA-9d	0.5571	-0.6989	1.8131	0.8420
UJA-144d vs UJA-9d	0.1974	-1.0586	1.4534	0.9999
UJA-165d vs UJA-9d	0.5558	-0.7002	1.8118	0.8438
UJA-180d vs UJA-9d	0.5254	-0.7306	1.7814	0.8803
UJA-C-180d vs UJA-9d	1.6644	0.2601	3.0686	0.0129
UJA-22d vs UJA-16d	-0.2743	-1.5302	0.9817	0.9981
UJA-58d vs UJA-16d	-0.0446	-1.3006	1.2114	1.0000
UJA-C-58d vs UJA-16d	0.5937	-0.6623	1.8497	0.7913
UJA-80d vs UJA-16d	0.2075	-1.0485	1.4635	0.9998
UJA-144d vs UJA-16d	-0.1522	-1.4082	1.1038	1.0000
UJA-165d vs UJA-16d	0.2062	-1.0498	1.4622	0.9998
UJA-180d vs UJA-16d	0.1758	-1.0802	1.4318	0.9999
UJA-C-180d vs UJA-16d	1.3148	-0.0895	2.7190	0.0779
UJA-58d vs UJA-22d	0.2296	-1.0264	1.4856	0.9995
UJA-C-58d vs UJA-22d	0.8680	-0.3880	2.1240	0.3460
UJA-80d vs UJA-22d	0.4817	-0.7742	1.7377	0.9234
UJA-144d vs UJA-22d	0.1220	-1.1340	1.3780	1.0000
UJA-165d vs UJA-22d	0.4804	-0.7756	1.7364	0.9245
UJA-180d vs UJA-22d	0.4500	-0.8060	1.7060	0.9475

UJA-C-180d vs UJA-22d	1.5890	0.1848	2.9932	0.0192
UJA-C-58d vs UJA-58d	0.6384	-0.6176	1.8943	0.7219
UJA-80d vs UJA-58d	0.2521	-1.0039	1.5081	0.9990
UJA-144d vs UJA-58d	-0.1076	-1.3636	1.1484	1.0000
UJA-165d vs UJA-58d	0.2508	-1.0052	1.5068	0.9991
UJA-180d vs UJA-58d	0.2204	-1.0356	1.4764	0.9997
UJA-C-180d vs UJA-58d	1.3594	-0.0449	2.7636	0.0626
UJA-80d vs UJA-C-58d	-0.3862	-1.6422	0.8698	0.9793
UJA-144d vs UJA-C-58d	-0.7459	-2.0019	0.5100	0.5387
UJA-165d vs UJA-C-58d	-0.3876	-1.6436	0.8684	0.9788
UJA-180d vs UJA-C-58d	-0.4180	-1.6739	0.8380	0.9661
UJA-C-180d vs UJA-C-58d	0.7210	-0.6832	2.1253	0.7112
UJA-144d vs UJA-80d	-0.3597	-1.6157	0.8963	0.9870
UJA-165d vs UJA-80d	-0.0013	-1.2573	1.2547	1.0000
UJA-180d vs UJA-80d	-0.0317	-1.2877	1.2243	1.0000
UJA-C-180d vs UJA-80d	1.1073	-0.2970	2.5115	0.2016
UJA-165d vs UJA-144d	0.3584	-0.8976	1.6144	0.9873
UJA-180d vs UJA-144d	0.3280	-0.9280	1.5840	0.9931
UJA-C-180d vs UJA-144d	1.4670	0.0627	2.8712	0.0363
UJA-180d vs UJA-165d	-0.0304	-1.2864	1.2256	1.0000
UJA-C-180d vs UJA-165d	1.1086	-0.2956	2.5128	0.2004
UJA-C-180d vs UJA-180d	1.1390	-0.2653	2.5432	0.1759

Table S12. Results of Tukey’s Post-hoc tests for mean differences in species richness between the UJA samples.

		95% confidence interval		
	Mean Difference	lower bound	upper bound	adjusted *p* vale
UJA-16d vs UJA-9d	-113.67	-275.12	47.79	0.3283
UJA-22d vs UJA-9d	-110.00	-271.46	51.46	0.3686
UJA-58d vs UJA-9d	-115.67	-277.12	45.79	0.3075
UJA-C-58d vs UJA-9d	-55.67	-217.12	105.79	0.9599
UJA-80d vs UJA-9d	-134.67	-296.12	26.79	0.1543
UJA-144d vs UJA-9d	-78.67	-240.12	82.79	0.7698
UJA-165d vs UJA-9d	-55.33	-216.79	106.12	0.9614
UJA-180d vs UJA-9d	51.33	-110.12	212.79	0.9757
UJA-C-180d vs UJA-9d	-127.67	-289.12	33.79	0.2016
UJA-22d vs UJA-16d	3.67	-157.79	165.12	1.0000
UJA-58d vs UJA-16d	-2.00	-163.46	159.46	1.0000
UJA-C-58d vs UJA-16d	58.00	-103.46	219.46	0.9490
UJA-80d vs UJA-16d	-21.00	-182.46	140.46	1.0000
UJA-144d vs UJA-16d	35.00	-126.46	196.46	0.9984

UJA-165d vs UJA-16d	58.33	-103.12	219.79	0.9473
UJA-180d vs UJA-16d	165.00	3.54	326.46	0.0427
UJA-C-180d vs UJA-16d	-14.00	-175.46	147.46	1.0000
UJA-58d vs UJA-22d	-5.67	-167.12	155.79	1.0000
UJA-C-58d vs UJA-22d	54.33	-107.12	215.79	0.9654
UJA-80d vs UJA-22d	-24.67	-186.12	136.79	0.9999
UJA-144d vs UJA-22d	31.33	-130.12	192.79	0.9993
UJA-165d vs UJA-22d	54.67	-106.79	216.12	0.9641
UJA-180d vs UJA-22d	161.33	-0.12	322.79	0.0503
UJA-C-180d vs UJA-22d	-17.67	-179.12	143.79	1.0000
UJA-C-58d vs UJA-58d	60.00	-101.46	221.46	0.9382
UJA-80d vs UJA-58d	-19.00	-180.46	142.46	1.0000
UJA-144d vs UJA-58d	37.00	-124.46	198.46	0.9975
UJA-165d vs UJA-58d	60.33	-101.12	221.79	0.9362
UJA-180d vs UJA-58d	167.00	5.54	328.46	0.0390
UJA-C-180d vs UJA-58d	-12.00	-173.46	149.46	1.0000
UJA-80d vs UJA-C-58d	-79.00	-240.46	82.46	0.7658
UJA-144d vs UJA-C-58d	-23.00	-184.46	138.46	0.9999
UJA-165d vs UJA-C-58d	0.33	-161.12	161.79	1.0000
UJA-180d vs UJA-C-58d	107.00	-54.46	268.46	0.4036
UJA-C-180d vs UJA-C-58d	-72.00	-233.46	89.46	0.8431
UJA-144d vs UJA-80d	56.00	-105.46	217.46	0.9585
UJA-165d vs UJA-80d	79.33	-82.12	240.79	0.7618
UJA-180d vs UJA-80d	186.00	24.54	347.46	0.0162
UJA-C-180d vs UJA-80d	7.00	-154.46	168.46	1.0000
UJA-165d vs UJA-144d	23.33	-138.12	184.79	0.9999
UJA-180d vs UJA-144d	130.00	-31.46	291.46	0.1847
UJA-C-180d vs UJA-144d	-49.00	-210.46	112.46	0.9820
UJA-180d vs UJA-165d	106.67	-54.79	268.12	0.4076
UJA-C-180d vs UJA-165d	-72.33	-233.79	89.12	0.8397
UJA-C-180d vs UJA-180d	-179.00	-340.46	-17.54	0.0225

Table S13. Results of Tukey’s Post-hoc tests for mean differences in Pielou’s evenness between the UJA samples

		95% confidence interval		
	Mean Difference	lower bound	upper bound	adjusted *p* vale
UJA-16d vs UJA-9d	0.0669	-0.1536	0.2873	0.9810
UJA-22d vs UJA-9d	0.0208	-0.1996	0.2413	1.0000
UJA-58d vs UJA-9d	0.0671	-0.1533	0.2876	0.9805

UJA-C-58d vs UJA-9d	0.2911	0.0706	0.5115	0.0046
UJA-80d vs UJA-9d	0.1021	-0.1184	0.3225	0.8088
UJA-144d vs UJA-9d	0.0363	-0.1841	0.2568	0.9998
UJA-165d vs UJA-9d	0.0644	-0.1560	0.2848	0.9852
UJA-180d vs UJA-9d	0.0929	-0.1275	0.3134	0.8756
UJA-C-180d vs UJA-9d	0.3710	0.1246	0.6175	0.0011
UJA-22d vs UJA-16d	-0.0460	-0.2664	0.1744	0.9987
UJA-58d vs UJA-16d	0.0003	-0.2201	0.2207	1.0000
UJA-C-58d vs UJA-16d	0.2242	0.0038	0.4446	0.0443
UJA-80d vs UJA-16d	0.0352	-0.1852	0.2556	0.9998
UJA-144d vs UJA-16d	-0.0305	-0.2509	0.1899	1.0000
UJA-165d vs UJA-16d	-0.0024	-0.2229	0.2180	1.0000
UJA-180d vs UJA-16d	0.0261	-0.1943	0.2465	1.0000
UJA-C-180d vs UJA-16d	0.3042	0.0577	0.5506	0.0089
UJA-58d vs UJA-22d	0.0463	-0.1741	0.2667	0.9986
UJA-C-58d vs UJA-22d	0.2702	0.0498	0.4906	0.0095
UJA-80d vs UJA-22d	0.0812	-0.1392	0.3017	0.9385
UJA-144d vs UJA-22d	0.0155	-0.2049	0.2359	1.0000
UJA-165d vs UJA-22d	0.0436	-0.1768	0.2640	0.9991
UJA-180d vs UJA-22d	0.0721	-0.1483	0.2925	0.9694
UJA-C-180d vs UJA-22d	0.3502	0.1038	0.5966	0.0022
UJA-C-58d vs UJA-58d	0.2239	0.0035	0.4443	0.0447
UJA-80d vs UJA-58d	0.0349	-0.1855	0.2553	0.9999
UJA-144d vs UJA-58d	-0.0308	-0.2512	0.1896	0.9999
UJA-165d vs UJA-58d	-0.0027	-0.2231	0.2177	1.0000
UJA-180d vs UJA-58d	0.0258	-0.1946	0.2462	1.0000
UJA-C-180d vs UJA-58d	0.3039	0.0575	0.5503	0.0090
UJA-80d vs UJA-C-58d	-0.1890	-0.4094	0.0314	0.1315
UJA-144d vs UJA-C-58d	-0.2547	-0.4751	-0.0343	0.0161
UJA-165d vs UJA-C-58d	-0.2266	-0.4471	-0.0062	0.0409
UJA-180d vs UJA-C-58d	-0.1981	-0.4185	0.0223	0.1003
UJA-C-180d vs UJA-C-58d	0.0800	-0.1664	0.3264	0.9709
UJA-144d vs UJA-80d	-0.0657	-0.2861	0.1547	0.9830
UJA-165d vs UJA-80d	-0.0377	-0.2581	0.1828	0.9997
UJA-180d vs UJA-80d	-0.0091	-0.2295	0.2113	1.0000
UJA-C-180d vs UJA-80d	0.2690	0.0225	0.5154	0.0258
UJA-165d vs UJA-144d	0.0281	-0.1923	0.2485	1.0000
UJA-180d vs UJA-144d	0.0566	-0.1638	0.2770	0.9939
UJA-C-180d vs UJA-144d	0.3347	0.0883	0.5811	0.0035
UJA-180d vs UJA-165d	0.0285	-0.1919	0.2489	1.0000
UJA-C-180d vs UJA-165d	0.3066	0.0602	0.5531	0.0083
UJA-C-180d vs UJA-180d	0.2781	0.0317	0.5245	0.0197

Reference:

1. Baron, D.; Palmer, C. D., Solubility of jarosite at 4–35 °C. *Geochim. Cosmochim. Acta.* **1996,** *60*, (2), 185-195.
2. Regenspurg, S.; Brand, A.; Peiffer, S., Formation and stability of schwertmannite in acidic mining lakes. *Geochim. Cosmochim. Acta.* **2004,** *68*, (6), 1185-1197.
3. Schwertmann, U.; Cornell, R. M., *Iron oxides in the laboratory: preparation and characterization*. John Wiley & Sons: 2008.
4. Schippers, A.; Jrgensen, B. B., Oxidation of pyrite and iron sulfide by manganese dioxide in marine sediments. *Geochim. Cosmochim. Acta.* **2001,** *65*, (6), 915-922.
5. Burton, E. D.; Johnston, S. G., Impact of silica on the reductive transformation of schwertmannite and the mobilization of arsenic. *Geochim. Cosmochim. Acta.* **2012,** *96*, 134-153.
6. Shahabi-Ghahfarokhi, S.; Åström, M.; Yu, C.; Lindquisit, T.; Djerf, H.; Kalbitz, K.; Ketzer, M., Extensive dispersion of metals from hemiboreal acid sulfate soil into adjacent drain and wetland. *Appl. Geochem.* **2022,** *136*.
7. Kelly, S. D.; Kemner, K. M.; Carley, J.; Criddle, C.; Jardine, P. M.; Marsh, T. L.; Phillips, D.; Watson, D.; Wei-Min, W. U., Speciation of uranium in sediments before and after in situ biostimulation. *Environ. Sci. Technol.* **2008,** *42*, (5), 1558-1564.
8. Ravel, B.; Newville, M., ATHENA, ARTEMIS, HEPHAESTUS: data analysis for X-ray absorption spectroscopy using IFEFFIT. *J. Synchrotron Radiat.* **2005,** *12*, (4), 537-541.
9. Templeton, D. H.; Zalkin, A.; Ruben, H.; Templeton, L. K., Redetermination and absolute configuration of sodium uranyl(VI) triacetate. *Acta. Crystallogr.* **2014,** *41*, (10), 1439-1441.
10. Fernandez-Martinez, A.; Timon, V.; Roman-Ross, G.; Cuello, G. J.; Ayora, C., The structure of schwertmannite, a nanocrystalline iron oxyhydroxysulfate. *Am. Mineral.* **2010,** *95*, (8-9), 1312-1322.
11. Basciano, L. C.; Peterson, R. C., Jarosite-hydronium jarosite solid-solution series with full iron site occupancy: Mineralogy and crystal chemistry. *Am. Mineral.* **2007,** *92*, (8-9), 1464-1473.
12. Locock, A. J.; Burns, P. C., The crystal structure of Triuranyl Diphosphate Tetrahydrate. *J. Solid State Chem.* **2002,** *163*, (1), 275-280.
13. Bargar, J. R.; Reitmeyer, R.; Lenhart, J. J.; Davis, J. A., Characterization of U(VI)-carbonato ternary complexes on hematite EXAFS and electrophoretic mobility measurements. *Geochim. Cosmochim. Acta.* **2000,** *64*, (16), 2737-2749.
14. Kelly, S. D.; Kemner, K. M.; Fein, J. B.; Fowle, D. A.; Yee, N., X-ray absorption fine structure determination of pH-dependent U-bacterial cell wall interactions. *Geochim. Cosmochim. Acta.* **2002,** *66*, (22), 3855-3871.
15. Högfors-Rönnholm, E.; Christel, S.; Lillhonga, T.; Engblom, S.; Österholm, P.; Dopson, M., Biodegraded peat and ultrafine calcium carbonate result in retained metals and higher microbial diversities in boreal acid sulfate soil. *Soil. Ecol. Lett.* **2020,** *2*, 120-130.
16. Herlemann, D. P.; Labrenz, M.; Jürgens, K.; Bertilsson, S.; Waniek, J. J.; Andersson, A. F., Transitions in bacterial communities along the 2000 km salinity gradient of the Baltic Sea. *ISME. J.* **2011,** *5*, (10), 1571-1579.
17. Hugerth, L. W.; Wefer, H. A.; Lundin, S.; Jakobsson, H. E.; Lindberg, M.; Rodin, S.; Engstrand, L.; Andersson, A. F., DegePrime, a program for degenerate primer design for broad-taxonomic-range PCR in microbial ecology studies. *Appl. Environ. Microbiol.* **2014,** *80*, (16), 5116-5123.
18. Straub, D.; Blackwell, N.; Langarica-Fuentes, A.; Peltzer, A.; Nahnsen, S.; Kleindienst, S., Interpretations of environmental microbial community studies are biased by the selected 16S rRNA (gene) amplicon sequencing pipeline. *Front. Microbiol.* **2020,** *11*, 550420.
19. Bolyen, E.; Rideout, J. R.; Dillon, M. R.; Bokulich, N. A.; Abnet, C. C.; Al-Ghalith, G. A.; Alexander, H.; Alm, E. J.; Arumugam, M.; Asnicar, F., Reproducible, interactive, scalable and extensible microbiome data science using QIIME 2. *Nat. Biotechnol.* **2019,** *37*, (8), 852-857.
20. Callahan, B. J.; McMurdie, P. J.; Rosen, M. J.; Han, A. W.; Johnson, A. J. A.; Holmes, S. P., DADA2: High-resolution sample inference from Illumina amplicon data. *Nat. Methods.* **2016,** *13*, (7), 581-583.
21. Klindworth, A.; Pruesse, E.; Schweer, T.; Peplies, J.; Quast, C.; Horn, M.; Glöckner, F. O., Evaluation of general 16S ribosomal RNA gene PCR primers for classical and next-generation sequencing-based diversity studies. *Nucleic. Acids. Res.* **2013,** *41*, (1), e1-e1.

22. Johnson, A.; Högfors-Rönnholm, E.; Engblom, S.; Österholm, P.; Åström, M.; Dopson, M., Dredging and deposition of metal sulfide rich river sediments results in rapid conversion to acid sulfate soil materials. *Sci. Total. Environ.* **2022,** *813*, 151864.
23. R Core Team, R: A Language and Environment for Statistical Computing R Foundation for Statistical Computing, Vienna, Austria. **2019**.
24. Oksanen, J.; Blanchet, F. G.; Friendly, M.; Kindt, R.; Legendre, P.; McGlinn, D.; Minchin, P. R.; O'hara, R.; Simpson, G.; Solymos, P., Vegan: community ecology package (version 2.5-6). *The Comprehensive R Archive Network* **2019**.
25. Wickham, H.; Averick, M.; Bryan, J.; Chang, W.; McGowan, L. D. A.; François, R.; Grolemund, G.; Hayes, A.; Henry, L.; Hester, J., Welcome to the Tidyverse. *J. Open Source Softw.* **2019,** *4*, (43), 1686.
26. Aitchison, J., The statistical analysis of compositional data. *J. R. Stat. Soc. B.* **1982,** *44*, (2), 139-160.
27. Salter, S. J.; Cox, M. J.; Turek, E. M.; Calus, S. T.; Cookson, W. O.; Moffatt, M. F.; Turner, P.; Parkhill, J.; Loman, N. J.; Walker, A. W., Reagent and laboratory contamination can critically impact sequence-based microbiome analyses. *BMC Biol.* **2014,** *12*, 1-12.
